# Synthetic DNA co-immunization with vaccine-aligned common consensus nucleoprotein and hemagglutinin protects mice against lethal influenza infection with a single immunization

**DOI:** 10.3389/fimmu.2025.1632121

**Published:** 2025-11-26

**Authors:** Ebony N. Gary, Abigail R. Trachtman, Dan Wang, Suman Bharti, Ying Ye, Nicholas J. Tursi, Martina Tomirotti, Jillian Eisenhauer, Jacqueline D. Chu, David Custodio Zegarra, Casey E. Hojecki, Micki Zheng, Jayamanna Wickramasinghe, David B. Weiner, Ami Patel

**Affiliations:** 1 The Vaccine and Immunotherapy Center, The Wistar Institute, Philadelphia, PA, United States; 2 Bioinformatics Facility, The Wistar Institute, Philadelphia, PA, United States; 3 Perelman School of Medicine, The University of Pennsylvania, Philadelphia, PA, United States; 4 Pharmacy and Biotechnology Department, University of Bologna, Bologna, Italy; 5 Vagelos Program in Life Sciences and Management, The University of Pennsylvania, Philadelphia, PA, United States

**Keywords:** influenza A virus, nucleoprotein, hemagglutinin, synthetic DNA, multivalent vaccine

## Abstract

**Introduction:**

There is an urgent need for influenza vaccine strategies that enhance protection against influenza virus drift and across different subtypes. The conserved viral nucleoprotein (NP) is the most abundant viral protein during replication, and a target for broadly protective cellular immune responses.

**Methods:**

Guided by annual WHO-recommended seasonal vaccine strains, we engineered synthetic DNA vaccine candidates encoding vaccine-aligned common consensus (VACC) immunogens designed to represent the immune diversity of seasonal H1N1 and H3N2 virus NP proteins (pVACC-NPH1; pVACC-NPH3).

**Results:**

Both pVACC-NPH1 and pVACC-NPH3 DNA vaccines induced robust cellular immune responses in mice, including the induction of durable responses. Immunization with a single dose of either DNA vaccine 14 days prior to lethal A/California/2009 H1N1 virus challenge provided protection against mortality. Single dose co-administration of pVACC-NPH3 with an HA-expressing DNA vaccine (pHAH1) and plasmid-encoded adjuvant pIL-12 afforded improved protection against morbidity and mortality in a high-dose challenge model.

**Discussion:**

These data highlight the potential of heterologous cellular immunity induced by engineered NP immunogens to complement HA-based approaches to significantly improve challenge outcomes.

## 1 Introduction

Seasonal influenza viruses infect approximately 1 billion people each year (1), causing respiratory illnesses across both hemispheres. An estimated 3–5 million of these cases result in severe illness, with 290-650, 000 deaths annually (2). Although yearly vaccination against circulating influenza A virus (IAV) H1N1 and H3N2, and B virus strains is recommended, genetic variation necessitates annual reformulation (3–6). Broad and universal influenza vaccines are urgently needed to protect from circulating and newly emerging influenza viruses. In addition to strategies that induce broadly protective antibodies against the viral surface hemagglutinin (HA) protein (7–10), synthetic immunogen approaches (11–14) that direct protective immunity to target highly conserved epitopes or proteins could provide important adjunctive protection to decrease pathogenesis and severe disease.

The influenza nucleoprotein (NP) is the most abundantly expressed protein during viral replication and the major component of the virion ribonucleoprotein complex (15). It plays a critical role in viral replication, involving organization of RNA packing, nuclear trafficking, vRNA transcription. and replication (16). NP is well-conserved within influenza subtypes (17–21), making it a promising target for inducing cellular immune responses. NP has been shown to induce robust CD8^+^ T cell responses in preclinical models (22, 23) and humans (24). Computational modeling of influenza isolates has revealed stretches of highly conserved amino acids within NP. Peptide vaccines based on such epitopes elicit robust CD8^+^ T cell responses and are protective against IAV challenge in mice (18). Epidemiological studies indicate that anti-NP CD8^+^ T cell immunity can contribute to protection from severe disease in humans (25). These data suggest that NP based therapies have the potential to elicit broad anti-influenza cellular immunity.

Synthetic plasmid DNA vaccines have advanced significantly over the past ten years, demonstrating robust induction of humoral and cellular immune responses (26). The first DNA vaccine received EUA for use in humans during COVID-19 (27) and several T cell-based DNA vaccines are being evaluated for infectious diseases and delivery of cancer neoepitopes (28) to elicit CD8+ T cell responses. Current inactivated vaccines elicit poor CD8^+^ T cell responses compared to live attenuated influenza vaccines (LAIV) (29–31). Although LAIV vaccines can induce CD8^+^ T cell responses, the master donor virus used to make all LAIVs contains the internal genes, including NP, of A/Ann Arbor/6/60 or A/Leningrad/17/57 H2N2 viruses and is thus mismatched to modern circulating strains. To this end, studies matching LAIV vaccines to currently circulating viruses can increase induction of CD8^+^ T cell responses (32). Building on this prior research, we hypothesized that plasmid DNA-encoded NP consensus immunogens could expand the breadth of protection, eliciting broad cellular immunity which could reduce IAV pathogenesis.

Here, we describe the design and evaluation of synthetic IAV-NP immunogens engineered based on WHO-recommended vaccine strains to induce robust anti-influenza cellular immunity *in vivo*. Two plasmid DNA-encoded vaccine-aligned common consensus (VACC) immunogens representing the NPs from seasonal A/H1N1 (VACC-NP^H1^) or A/H3N2 (VACC-NP^H3^) viruses induced robust cellular immune responses, with both independently providing single dose protection against mortality in mice intranasally challenged with an A/California/2009 virus. We delivered these antigens alone or in combination with plasmid-encoded IL-12 (pIL-12) which has been demonstrated to enhance cellular responses to DNA antigens in mice, non-human primates (33, 34), and humans in clinical trials (35–37). Heterologous pVACC-NP^H3^ combination with plasmid-encoded hemagglutinin (HA) from H1N1 A/California/07/2009 (pHA^H1^) and pIL-12 afforded complete protection from IAV-associated morbidity and mortality, further highlighting the potential for synthetic VACC-NP^X^ candidates to reduce pathogenesis and provide immune protective benefit across IAV subtypes.

## 2 Methods

### 2.1 Plasmid design

The amino acid sequences for NP proteins from WHO recommended H1N1 and H3N2 vaccine strains selected from 2000–2019 vaccine strains (38) were downloaded from the GISAID.org database. H1N1 NP accession #: A/New Caledonia/20/1999 (EPI ISL 649), A/Solomon Islands/3/2006 (EPI224787), A/Brisbane/59/2007 (EPI ISL 154495), A/California/07/2009 (EPI ISL 391380), A/Michigan/45/2015 (EPI ISL 199532)A/Brisbane/02/2018 (EPI ISL 344858), A/Wisconsin/588/2019 (EPI ISL 404527), A/Hawaii/70/2019 (EPI ISL 397028). H3N2 NP accession #: A/Moscow/10/1999 (EPI ISL 2695), A/Fujian/411/2002 (EPI ISL 107711), A/California/7/2004 (EPI ISL 113070), A/Wisconsin/67/2005 (EPI ISL 154528), A/Brisbane/10/2007 (EPI ISL 176458), A/Perth/16/2009 (EPI ISL 176456), A/Victoria/361/2011 (EPI ISL 101506), A/Switzerland/9715293/2013 (EPI ISL 166310), A/Hong Kong/4801/2014 (EPI ISL 233740), A/Singapore/INFIMH-16-0019/2016 (EPI2397166), A/Kansas/14/2017 (EPI ISL 292575), A/Hong Kong/45/2019 (EPI ISL 347938). H1NP or H3NP vaccine-consensus designs were constructed through sequence alignment analysis in MEGA 11.0.10 (39) using ClustalW alignment and an unrooted phylogenetic tree was generated using the maximum-likelihood method, with maximum parsimony (40). Pairwise distances were calculated in MEGA. 11.0.10. Sequence identity visualization was performed in Treeviewer (41). Additional alignment of sequences were performed in Geneious Prime (version 2023.2.1). mRNA expression was confirmed by qPCR using the following primers: NP^H1^ Forward: (5’-GATCTCTGTGCAGCCTACCT-3’), Reverse (5’-ATCACTTCTGTGCGCATGTC-3’), NP^H3^ Forward (5’-TCTGCCTTTGACGAGAGGAG-3’), Reverse (5’-CCGCCAGATTCTCCTGATCT-3’), and mouse GAPDH (NM_008084) CAT#: MP205604) Forward (5’-CATCACTGCCACCCAGAAGACTG-3’), Reverse (5’-ATGCCAGTGAGCTTCCCGTTCAG3’), all will amplicon sizes of 120 base pairs. Analysis was performed using comparative delta-delta CT analysis. DNA plasmid encoding the full-length codon-optimized, HA protein of A/California/07/2009 H1N1pdm09 cloned into the pVax1 vector (pHA^H1^) was previously described in (42). The plasmid-encoded adjuvant mouse interleukin 12 (IL12) has been previously described in (43).

### 2.2 Cell lines and virus propagation

Influenza A Virus, A/California/07/2009 NYMC X-179A H1N1pdm09 (Ca09-X179A) (IRR catalog: FR-246), was obtained through the International Reagent Resource, Influenza Division, WHO Collaborating Center for Surveillance, Epidemiology and Control of Influenza, Centers for Disease Control and Prevention, Atlanta, GA, USA. This is a reassortant virus with the HA, NA, and PB1 genes from H1N1pdm09 and remaining genes from A/Puerto Rico/8/1934. MDCK-SIAT1 cells (Sigma Cat# 5071502) were maintained in Minimum Essential Medium (Eagle’s) (Corning Cat # MT10009CV) with 1% Penicillin/Streptomycin (Gibco Cat #15140122), and 2% fetal bovine serum (Peak Cat #PS-FB4). For virus propagation, cell monolayers were infected with MOI 0.001 of Ca09-X179A in the presence of 2 µg/mL TPCK-treated Trypsin (ThermoFisher Cat# 20233) and maintained with 1% Pen/Strep, 0.3% bovine serum albumin (Gibco Cat # 15260037) for 3 days. Virus was collected and ultracentrifuged on a sucrose gradient to prepare mouse challenge stocks. Challenge stocks were titered by determining the 50% tissue culture infectious dose (TCID50) on MDCK-SIAT1 cells and an initial mouse 50% lethal dose (LD50) experiment was performed to determine the minimum infectious dose for challenge.

### 2.3 Animals, immunization, and challenge

C57BL/6J (Stock # 000664) and DBA/2J (Stock # 000671) female mice were purchased from the Jackson Laboratory and were housed in the Wistar Institute Animal Facility. All procedures were done in accordance with the guidelines from the Wistar Institute Animal Care and Use Committee. Between 2 μg to 10 μg of DNA plasmid encoding the VACC-NP^H1^ or VACC-NP^H3^ or full length HA DNA (pHA^H1^) (42) with or without a DNA plasmid encoding for the molecular adjuvant IL-12, in 30 μL water was injected in the tibialis anterior (TA) muscle. Delivery was immediately followed with two 0.1 Amp electric constant current square-wave pulses by the CELLECTRA-3P electroporation device (Inovio Pharmaceuticals) to increase transfection efficiency. Immunized or naive DBA/2J mice were intranasally infected with 10 LD50 or 100 LD50 of Ca09-X179A respectively in 50 µl MEM Eagle’s (without antibiotics). Mice were then monitored for the subsequent 21 days, for weight loss and mortality. Any mouse reaching 80% of their original body weight was considered to have reached humane endpoint and was subsequently euthanized. A subset of mice (n=3 per group) was euthanized on day 6 post infection, and lungs were collected for histopathological analysis. The vaccine and challenge schedules are indicated in each figure.

### 2.4 Western blot

HEK293T cells (ATCC Cat# CRL-3216) were cultured in DMEM medium with 10% FBS at 37 °C/5% CO2 condition and transfected with pDNA using Lipofectamine 3000 transfection reagent (Thermo Fisher Scientific Cat# L300000) following the manufacturer’s protocol. Forty-eight hours later, supernatant and cell lysates were harvested using 1x cell lysis buffer (Cell signaling Cat# 9803). Proteins were separated on a 4–12% BIS-TRIS gel (Thermo Fisher Scientific Cat# NP0322BOX), then following transfer, blots were incubated with an anti-NP monoclonal antibody (Thermo Fisher Cat# PA5-32242), then visualized with horseradish peroxidase (HRP)-conjugated anti-rabbit IgG (Sigma Cat# SAB3701359).

### 2.5 Peptide reagents

Individual antigen-matched 15mer peptides with 11mer overlaps were synthesized (Genscript, Piscataway, NJ) for NP^H1^ and NP^H3^. Peptides were resuspended as a single peptide pool for flow cytometry, four peptide pools for ELISPOT, or 23 peptides per pool for epitope mapping. Individual NP peptides and pool information is listed in **Supplementary Table S1**. HA peptide pools are as previously described (42). All pools were resuspended in dimethyl sulfoxide (DMSO).

### 2.6 Flow cytometry

Immunized mice were euthanized, and spleens and lungs were harvested and stored in RPMI 1640 media (Invitrogen Cat# 11875093) supplemented with 10% FBS and 1% Penicillin/Streptomycin (R10). Spleens were processed to single-cell suspension and red blood cells were removed by ACK lysing buffer (Gibco Cat# A1049201). Lungs were processed using the lung dissociation kit/GentleMACS system (Miltenyi Cat# 130-095-927) according to manufacturer’s instruction. Red blood cells were removed by ACK lysing buffer (Gibco Cat# A1049201), and single cells isolated via density gradient centrifugation using lymphosep (MP Biomedicals Cat#: 0916922-CF). Cells were then filtered and counted before being plated for flow cytometry. Cells (1, 000, 000 per well) were seeded in 100 μL of R10 and stimulated with NP^H1^, the NP^H3^, or Ca09 HA peptide pools (5 μg/mL per peptide final concentration) in the presence of Protein Transport Inhibitor (eBioscience, San Diego, CA, USA Cat# 00-4980-03). R10 alone and cell Stimulation Cocktail containing phorbol 12-myristate 13-acetate (PMA) and ionomycin (500X, eBioscience, San Diego, CA, USA Cat# 00-4970-93) in R10 were used as negative and positive controls, respectively. Plates were incubated for 6 h at 37 °C with 5% CO2. After stimulation, cells were stained with LIVE/DEAD zombie aqua for viability. CD3, CD4, CD8, TNF-α, IFNγ, and IL-2 fluorochrome conjugated antibodies (BioLegend) were used for surface and intracellular staining. The samples were run on a BD FACSymphony™ A5 SE flow cytometer (BD Biosciences) and analyzed in FlowJo software. Gates were set using fluorescence minus one (FMO) for each stain. Data was exported and analyzed in GraphPad Prism 10.

### 2.7 ELISpot

Isolates splenocytes and lung lymphocytes (pulmocytes) were subjected to IFNγ ELISpot assay according to the manufacturer’s instructions (Mabtech Cat# 3321-4APW-10). Briefly, plates were washed four times with sterile PBS and blocked with R10 media for two hours. Splenocytes from each animal were seeded in duplicate wells with 200, 000 cells per well in 100µL R10. Cells were stimulated with NP^H1^, NP^H3^ or HA^H1^ peptide pools (5µg/ml per peptide). The peptide pools and matrix peptide pools are listed in **Supplementary Table S1**. Negative and positive controls were stimulated with DMSO or PMA/ionomycin respectively. Plates were incubated at 37 °C in 5% CO2 for 18 hours and were then developed following the manufacturer’s protocol. Plates were scanned and counted using the Mabtech IRIS™ FluoroSpot/ELISpot reader.

### 2.8 Histopathology and immunohistochemistry

Whole murine lungs were collected into 10% buffered neutral-buffered formalin for routine histopathological processing. Formalin fixed tissues were paraffin embedded and 4 μm sections were cut and routinely stained with Hematoxylin and Eosin (H&E). Immunohistochemical detection was performed on 4 μm tissue sections using a polyclonal antibody against the IAV nucleoprotein (anti-NP)(Thermo Fisher Cat# PA5-32242). Whole slides were scanned using a Hamamatsu Nanozoomer S60 slide scanner and analyzed using NDP.view 2. Scale bars equal 2.5 mm on whole slide lung images and 50 µm on lung section images.

### 2.9 RNA-seq

Formalin-fixed paraffin-embedded (FFPE) lung tissue scrolls of 10µM thickness were used for total RNA extraction. Total RNA was quantitated using the Qubit 2.0 Fluorometer (Thermo Fisher, Waltham, MA) and quality of RNA was assessed using the 4200 Tapestation (Agilent, Santa Clara, CA). Libraries for differential gene expression studies were prepared using the Quant Seq 3’ mRNA-Seq V2 Library Prep Kit FWD (Lexogen, Vienna, Austria) as per manufacturer’s instructions starting with an input of 350ng of RNA and 16 cycles of final PCR amplification. Overall library size was determined using the 4200 Tapestation and libraries were quantitated using the Qubit 2.0 Fluorometer. Libraries were pooled and Next Generation Sequencing with a single-end 76 bp run length was done on the Hiseq 1000 (Illumina, San Diego, CA). A minimum of 10M reads per sample was acquired for each sample. Using Cutadapt (44), we removed adapters and polyA in each sample, followed by alignment to the mm10 genome using Bowtie2 within the RSEM pipeline (v1.3.3). Only reads mapping to coding regions were retained. Raw counts and TPM values were generated for downstream analyses. Differential gene expression analysis was conducted using DESeq2 (v1.38.0). Genes with fewer than 10 raw counts were excluded, and DEGs were identified using FDR < 5% and |log2 fold change| ≥ 3. Functional enrichment was performed using Gene Ontology, KEGG pathways, and Ingenuity Pathway Analysis (IPA). Computational analyses were conducted on a Linux-based high-performance computing environment with tools Bowtie2 (v2.4.5), RSEM (v1.3.3), DESeq2 (v1.38.0), and IPA. Inhibited and activated pathway analysis is included as **Supplementary Tables S2**-**S5**, **S6**).”

### 2.10 Software and statistical analysis

Data was represented in GraphPad Prism version 10. All sequence alignments were determined in MEGA 11.0.10 (39) and Treeviewer (41), flow cytometry data was analyzed using FlowJo version 10.10.0. Image slides were scanned using a Hamamatsu Nanozoomer S60 slide scanner and analyzed using NDP.view vs2. Details on statistical analysis are included in the legend for each figure. The p-value significance is indicated as follows: *p<0.05, **p<0.01, ***p<0.001, ****p<0.0001, and comparisons are not significant (ns), unless otherwise denoted.

### 2.11 Data and code availability

The published article includes all data sets generated or analyzed during this study. Sequencing data was submitted to NCBI GEO database under accession number GSE306862.

## 3 Results

### 3.1 Design and expression of VACC-NP^X^ immunogens

**Figure 1:**
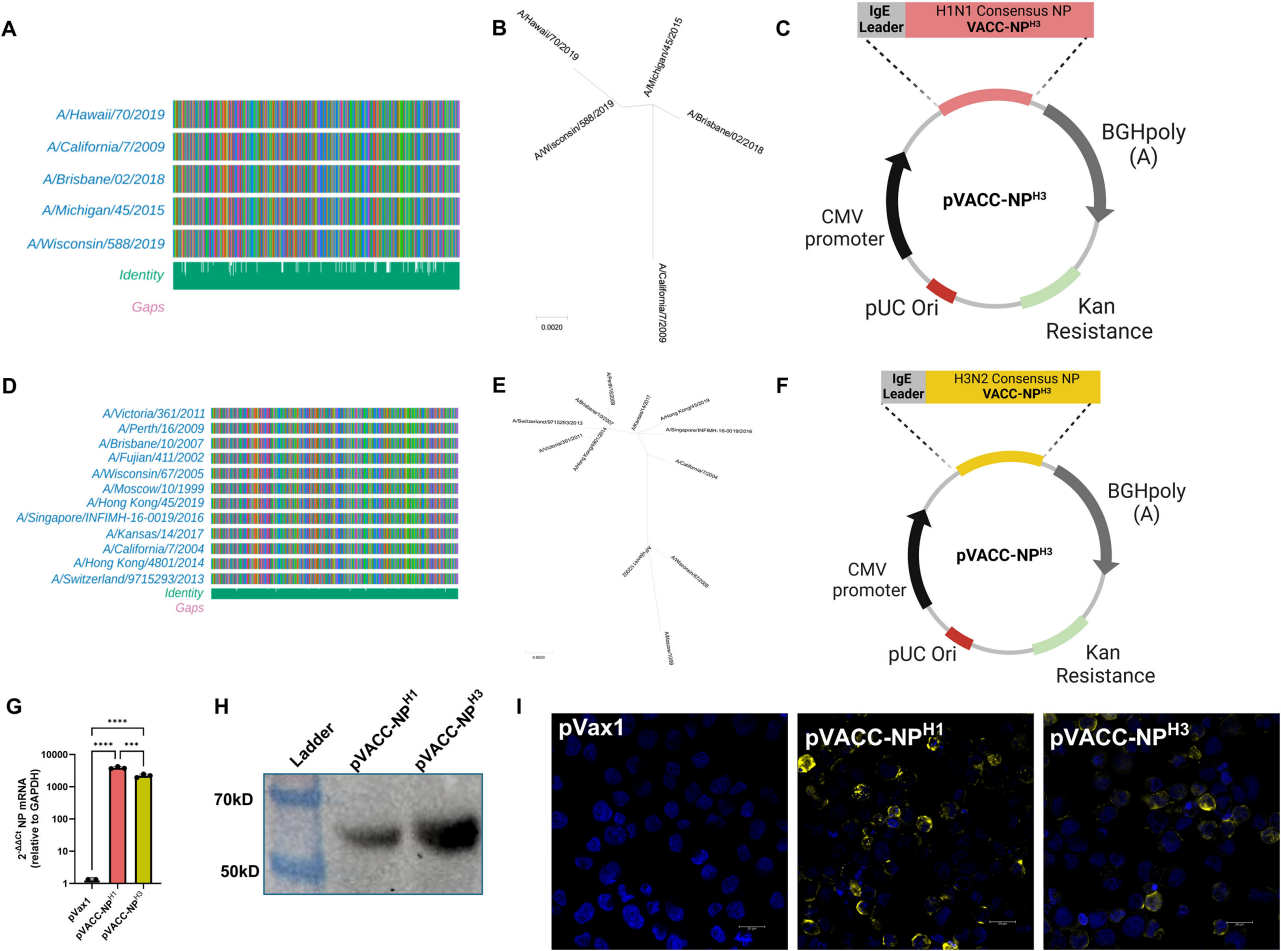
Design and *in vitro* expression of VACC-NP^X^ immunogens. NP amino acid alignments of seasonal A/H1N1 post-H1N1pdm09 and A/H3N2 vaccine strains (GISAID.org), **(A, D)**. Unrooted phylogenetic trees for A/H1N1 post-H1N1pdm09 and A/H3N2 vaccine strains **(B, E)**. Plasmid maps of pVACC-NP^H1^ and pVACC-NP^H3^ synthetic DNA constructs **(C, F)**. mRNA expression of VACC-NP^X^ by quantitative PCR following *in vitro* transfection **(G)**. Western blot of pVACC-NP^H1^ and pVACC-NP^H3^ HEK29T supernatants probed for anti-IAV-NP **(H)**. Immunofluorescence staining of HEK293T cells transfected with pVACC-NP^X^ plasmids and stained for IAV-NP **(I)**. Data are representative of two independent transfection experiments. Symbols **(G)** represent duplicate assays of three separate wells. ***p<0.001, ****P<0.0001 by Kruskal-Wallis ANOVA.

NP amino acid sequences for annual seasonal A/H1N1 vaccines strains were obtained from GISAID.org (**Supplementary Figures S1**, **S2**) and aligned to produce unrooted phylogenetic trees (**Supplementary Figure S1B**). Analysis highlighted a >10% amino acid distance between pre-2009 and post-H1N1pdm2009 viruses consistent with the major antigenic shift caused by introduction of a classical swine NP into the A/H1N1pdm09 lineage viruses (45, 46). We therefore focused our design on contemporary H1N1 viruses and generated a single vaccine-aligned consensus construct (VACC) design, using sequence alignments and phylogenetic analysis to weigh amino acids towards post-H1N1pdm2009 NPs (**Figures 1A, B**). VACC amino acid sequences were codon-optimized for mammalian expression and subcloned into the pVax1 plasmid DNA backbone to generate the pVACC-NP^H1^ construct (**Figure 1C**). Similarly, A/H3N2 vaccines strains were obtained from GISAID.org (**Supplementary Figures S1**, **S3**). A VACC-NP^H3^ construct was designed based on sequence alignments and phylogenetic analysis (**Figures 1D, E**) and cloned into the pVax1 backbone to generate the pVACC-NP^H3^ construct (**Figure 1F**). The overall pairwise distances were determined to be <0.2% for post-H1N1pdm09 IAV-NP^H1^ and <1.1% for IAV-NP^H3^. We confirmed mRNA expression of VACC-NP^X^ via quantitative PCR (**Figure 1G**) following *in vitro* transfection. Protein expression in transfected HEK 293cells was confirmed via supernatant western blot (**Figure 1H**), and immunofluorescence staining of IAV-NP (**Figure 1I**). Together these data demonstrate that the consensus alignment approach generates novel synthetic molecules that express *in vitro* and are detected by commercial anti-NP antibodies.

### 3.2 A single immunization with DNA-encoded pVACC-NP^X^ vaccines induces strong cellular responses and supports protection from influenza-associated morbidity and mortality *in vivo*

**Figure 2:**
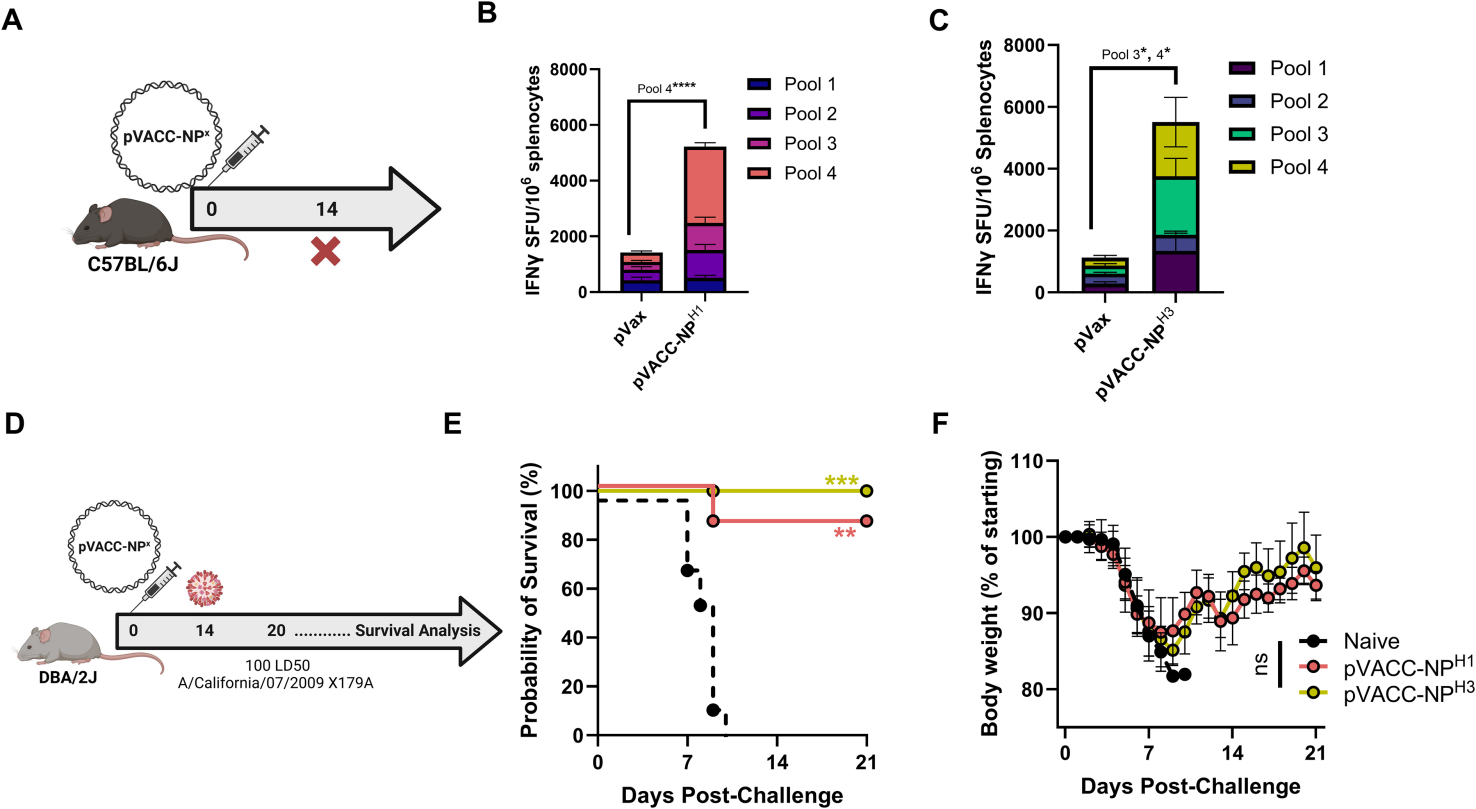
A single immunization with VACC-NP^X^ constructs is immunogenic and protects against mortality in an H1N1pdm09 mouse infection model. C57BL/6J mice were immunized with 10µg of pVACC-NP^X^ plasmids and euthanized fourteen days post-immunization for cellular analyses **(A)**. IFNγ spot-forming units (SFUs) in spleens following stimulation with H1NP peptides or H3NP peptides (n=5 mice per group) **(B, C)**. DBA/2J mice received a single administration of the pVACC-NP^H1^ or pVACC-NP^H3^ synthetic DNA vaccines (10µg, n=10 mice/group). After 14 days, the mice were intranasally challenged with 10 LD50 of H1N1 Ca09-X179A and monitored daily until day 21 post-challenge. On day 6 post-infection, a subset of mice (n=3) was euthanized lungs were collected and processed for histopathological analyses **(D)**. Survival probability **(E)**. Weight loss as percent of starting weight **(F)**. Hematoxylin and eosin staining **(G)**, and IAV-NP immunohistochemistry staining **(H)** of lung sections from representative mice at 6 days post-infection. Scale bars equal 2.5 mm on whole slide lung images. Data are representative of two independent experiments with n=5/group **(A–C)** and n=10/group **(D–H)**. Bars represent group means and error bars represent SEM **(B, C)**. Lines Symbols represent group averages; bars represent SD **(F)**. *p<0.05, **p<0.01, ***p<0.001, ****p<0.0001 by Two-way ANOVA **(B, C)**, Dunnett’s multiple comparison **(F)**, or Mantel-Cox Log-rank test **(E)**.

An initial dosing study in C57BL/6J mice was performed to assess the immunogenicity of 10 µg and 25 µg of the pVACC-NP^H3^ plasmid following a two-dose injection regimen. Both doses induced robust IFNγ spot-forming units (SFU) in spleens (**Supplementary Figure S4**). The difference between the two doses was not statistically significant, therefore we selected the lower 10 µg dose for evaluation as a single immunization regimen for both pVACC-NP^H1^ and pVACC-NP^H3^ immunogens. C57BL/6J mice were immunized once with 10 µg of pVACC-NP^H1^ or pVACC-NP^H3^ immunogens and cellular responses were evaluated by ELISpot assay fourteen days later (**Figure 2A**). pVACC-NP^H1^ induced significant NP^H1^-specific IFNγ SFU in the spleens of immunized mice as compared to empty plasmid (pVax1) immunized controls (**Figure 2B****),**. Similarly, pVACC-NP^H3^ resulted in significant induction of NP^H3^-specific IFNγ responses (**Figure 2C**). These data demonstrate that pVACC-NP^X^ constructs induce strong cellular immunity *in vivo*.

To evaluate the protective efficacy of these constructs, we used the DBA/2J mouse model of wild-type IAV challenge. DBA/2J mice were immunized once with 10 µg of pVACC-NP^H1^, pVACC-NP^H3^, or left unimmunized (naïve), and challenged fourteen days later with 10 LD50 of H1N1 Ca09-X179A (**Figure 2D**). Strikingly, we observed 90% survival among receiving pVACC-NP^H1^ and 100% survival among pVACC-NP^H3^ immunized animals, while all naïve animals succumbed to infection (**Figure 2E**). Despite surviving the challenge, all pVACC-NP^X^ immunized animals displayed weight loss similar to that observed in naïve animals (**Figure 2F**), and this was reflected by H&E staining of lungs harvested 6 days post-infection (**Figure 2G**). Dense cellular infiltrates were observed in the lungs of naive and pVACC-NP^H1^ immunized animals. pVACC-NP^H3^ immunized animals displayed decreased cellular infiltrates and increased airway space (**Figure 2G**). Similarly, when sections were stained for H1N1 NP antigen, naïve animals displayed significant NP-positive staining throughout their lungs. pVACC-NP^H1^ immunized animals had decreased NP antigen and only minimal staining was observed in the lungs of pVACC-NP^H3^ immunized mice (**Figure 2H**). Together, these data highlight the potential for a VACC-NP^X^ immunogen to provide benefit against disease and death following a single immunization.

### 3.3 Epitope mapping of VACC-NP^X^-induced cellular responses

A matrix system was used to organize vertical and horizontal peptide pools to identify immunodominant epitopes following immunization with either pVACC-NP^H1^ and pVACC-NP^H3^ (**Supplementary Table S1**). NP vaccines were co-formulated with gene-encoded adjuvant pIL-12, previously reported to enhance cellular (35, 37) and humoral (47) responses in humans and in preclinical models (33, 34, 48, 49) (**Supplementary Figure S5A**). Using this matrix format enables higher throughput identification of epitopes with limited samples (**Supplementary Table S1**). Immunodominant epitopes are identified if they demonstrate strong IFNγ responses in one vertical pool and one horizontal pool in the matrix. The peptides are then determined at the intersection of these two pools. In this way, we identified linear peptides ASNENVETM among NP^H1^ peptides **Supplementary Figures S5B, C**) and ASNENMDNM among NP^H3^ peptides (**Supplementary Figures S5D, E**), consistent with those described for murine H2-Db in the literature (50, 51). pVACC-NP^H3^ also elicited strong responses to the SAAFEDLRLLSFIRG peptide reported by *Lambe et al.* (51). Importantly, stimulation with the overlapping peptide pools containing the identified immunodominant epitopes (pool 4 for pVACC-NP^H1^ and pools 3 and 4 for pVACC-NP^H3^) resulted in significant increases in IFNγ secretion from immunized mice (**Figures 2A, B**). These data demonstrate that the pVACC-NP^X^ constructs can elicit responses consistent with previously identified epitopes, as well as can expand unique responses.

### 3.4 pVACC-NP^X^ antigens are amenable to co-delivery with HA immunogens

**Figure 3:**
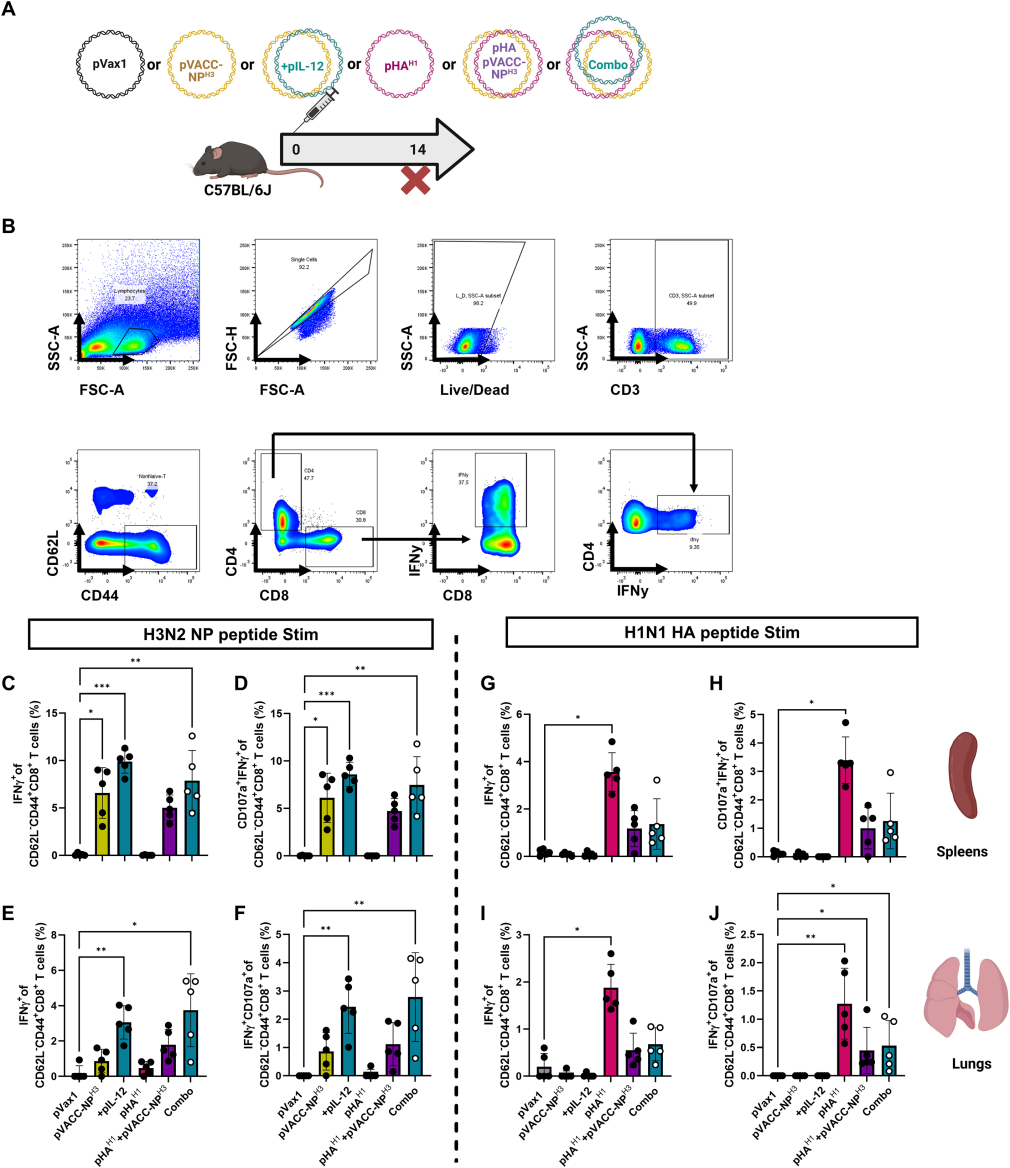
pVACC-NP^x^ immunogens are amenable to co-delivery with HA immunogens and gene-encoded adjuvant pIL-12. **(A)** C57BL/6J mice were immunized once with one of the following formulations: 12.5μg of empty plasmid vector (pVax1); 10μg of pVACC-NP^H3^ alone; pVACC-NP^H3^ plus 0.5μg of plasmid-encoded mouse IL-12 (+pIL-12); 2μg of plasmid-encoded A/California/07/2009 HA (pHA^H1^) alone; pVACC-NP^H3^ plus pHA^H1^; or a combination of pHA^H1^, pVACC-NP^H3^, and pIL-12 (Combo). Mice were euthanized fourteen days post-immunization for cellular analyses. **(B)** Gating strategy for intracellular cytokine staining using IFNγ^+^ splenocytes as an example. Cytokine positive CD4^+^ or CD8^+^ T cells were gated from single/live/CD3^+^/CD62L^-^/CD44^+^ cells. IFNγ^+^**(C)** and IFNγ^+^CD107^+^ effector CD8+ T cells **(D)** in spleens following stimulation with H3 NP peptides. IFNγ^+^**(E)** and IFNγ^+^CD107^+^ effector CD8+ T cells **(F)** in lungs following stimulation with H3 NP peptides. IFNγ^+^**(G)** and IFNγ^+^CD107^+^ effector CD8+ T cells **(H)** in spleens following stimulation with H1N1 HA peptides. IFNγ^+^**(I)** and IFNγ^+^CD107^+^ effector CD8+ T cells **(J)** in lungs following stimulation with H1N1 HA peptides. Data are representative of one independent experiment with n=5/group. Symbols represent individual animals, bars represent the group mean, and error bars represent SD. *p<0.05, **p<0.01, ***p<0.001, ****P<0.0001 by Kruskal-Wallis ANOVA.

Current seasonal influenza vaccines are either inactivated virus, live attenuated virus, or recombinant protein vaccines, driving primarily HA-directed antibody responses. We hypothesized that a synthetic VACC-NP^HX^ immunogen can provide adjunctive protection when administered in combination with an HA vaccine. Both pVACC-NP^H1^ and pVACC-NP^H3^ constructs induced similar immunogenicity and protective efficacy however pVACC-NP^H3^ demonstrated reduced lung pathogenesis following infection. Thus, we selected the heterologous pVACC-NP^H3^ for evaluation alone and in combination with a pHA^H1^ antigen. C57BL/6J mice were immunized once with one of the following formulations: 12.5μg of empty plasmid vector (pVax1); 10μg of pVACC-NP^H3^ alone; pVACC-NP^H3^ plus 0.5μg pIL-12; 2μg pHA^H1^ alone; pVACC-NP^H3^ plus pHA^H1^; or a combination of pHA^H1^, pVACC-NP^H3^, and pIL-12 (Combo). Mice were euthanized fourteen days post-immunization and cellular responses were quantified by intracellular cytokine staining (ICS) (**Figures 3A, B**).

Following NP^H3^ peptide stimulation, we observed statistically significant increases in IFNγ^+^ CD8^+^ effector cells in the spleens of animals immunized with pVACC-NP^H3^ alone, those co-immunized with pVACC-NP^H3^ and pIL-12, or those immunized with the combination of pVACC-NP^H3^, pIL-12, and pHA^H1^(Combo), as compared to those receiving empty plasmid control (pVax1) (**Figure 3C**). We similarly observed statistically significant increases in the frequency of CD107α^+^IFNy^+^ effector CD8^+^ T cells among these mice as compared to pVax1-immunized controls (**Figure 3D**). Cellular responses were assayed from lungs as it is the primary site of influenza infection and replication. We observed statistically significant increases in IFNγ^+^ CD8^+^ effector cells among isolated pulmocytes of animals immunized with pVACC-NP^H3^ alone, those co-immunized with pVACC-NP^H3^ and pIL-12, or those immunized with the combination of pVACC-NP^H3^, pIL-12, and pHA^H1^ (Combo), as compared to those receiving pVax1 (**Figure 3E**). We also observed statistically significant increases in the frequency of CD107α^+^IFNy^+^ effector CD8^+^ T cells among the pulmocytes of these mice as compared to pVax1-immunized controls (**Figure 3F**). In both the spleens and lungs, the addition of pIL-12 to pVACC-NP^H3^ trended toward increased IFNγ secretion compared to NP alone, but did not meet statistical significance.

Splenocytes stimulated with matched HA peptides demonstrated increased effector function among all groups receiving pHA^H1^, however only animals receiving pHA^H1^ alone had statistically significant increases in frequencies of IFNγ^+^ (**Figure 3G**) and CD107α^+^IFNy^+^ (**Figure 3H**) effector CD8^+^ T cells compared to those immunized with empty vector. Among pulmocytes, we observed trends toward increased effector function among all groups receiving pHA^H1^ with statistically significant increases in IFNy^+^ (**Figure 3I**), and CD107α^+^IFNy^+^ (**Figure 3J**) effector CD8^+^ T cell frequencies of animals receiving pHA^H1^ only, compared to those immunized with empty vector. Mice receiving pVACC-NP^H3^ or pVACC-NP^H3^ plus pIL-12 did not respond to HA peptide stimulation, highlighting the specificity of these vaccines. Lower responses were observed in the pHA^H1^+ pVACC-NP^H3^ group, suggesting potential interference when both antigens are co-delivered. Overall, these data suggest that combination delivery of pVACC-NP^X^ antigens with HA antigens can elicit robust NP-directed cellular responses in both the periphery and mucosa.

### 3.5 Heterologous pVACC-NP^H3^ enhances protective efficacy of the pHA^H1^ DNA vaccine against IAV H1N1pdm09 challenge

In the DBA/2J mouse model, a single immunization with pHA^H1^ alone induces complete protection against morbidity and mortality from a 10 LD_50_ homologous Ca09-X179A challenge (52). At the higher challenge inoculum of 100 LD_50_ mice immunized with 10 µg pHA^H1^ are completely protected from death but display significant weight loss before recovering (**Supplementary Figures S6A-C**). However, animals receiving either 1 µg or 0.5 µg of pHA^H1^ succumb to infection (**Supplementary Figure S6B**) and display significant weight loss (**Supplementary Figure S6C****).** This sub-protective model was next used to evaluate the protective efficacy following combination delivery of the pVACC-NP^H3^ and pHA^H1^ vaccines.

**Figure 4:**
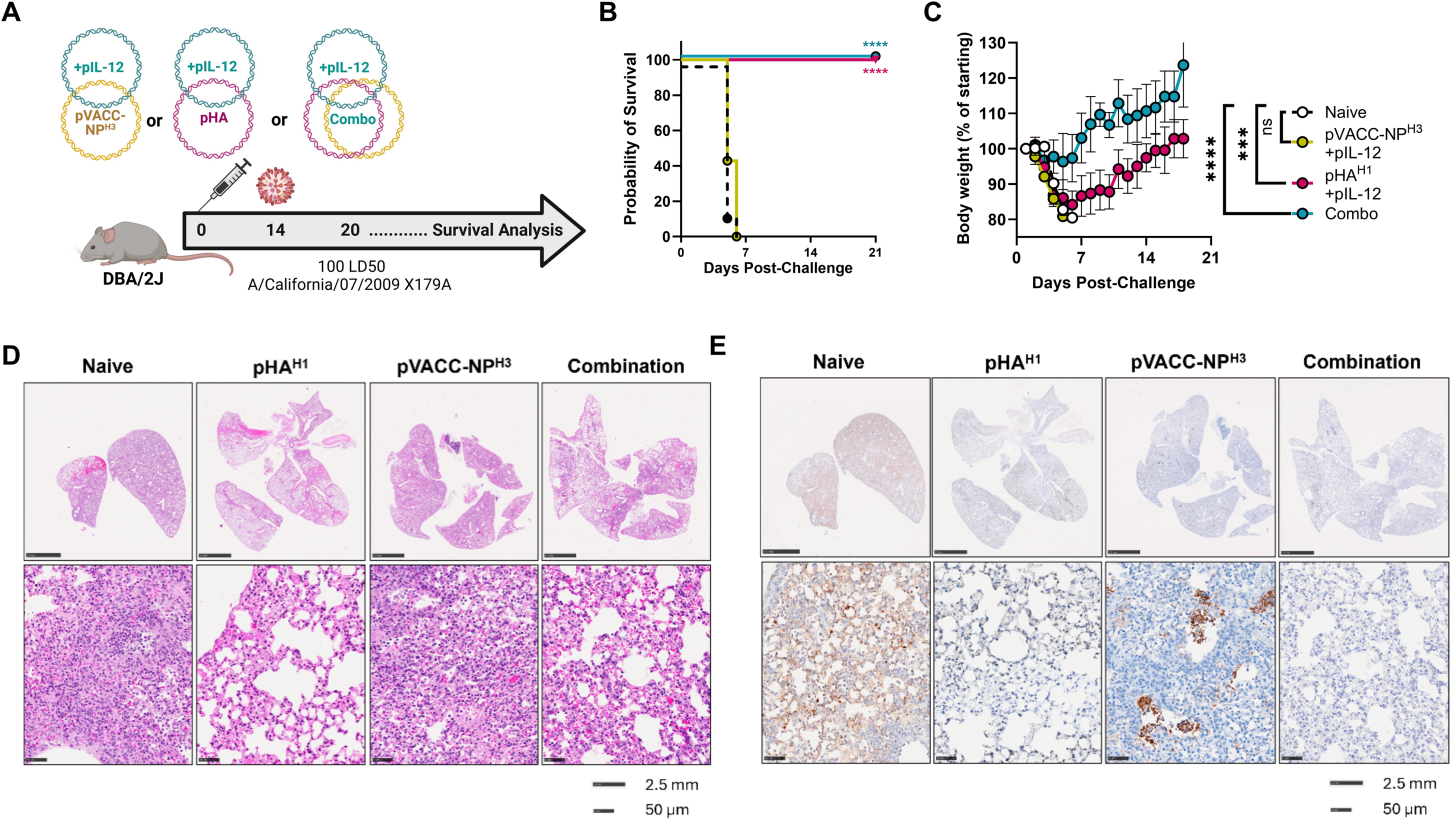
HA and NP combination improves protection from IAV induced morbidity DBA/2J mice were immunized once with 10μg of pHA and 0.5μg of pIL-12, 10μg pVACC- NP ^H3^ and 10μg of pIL-12, or co-immunized with pHA^H1^, pVACC-NP^H3^, and 0.5μg of IL-12 (Combo), and challenged with 100 LD50 Ca09-X179A virus 14 days later. Lungs were collected from 3 representative animals per group 6 days post-challenge and the remaining animals were monitored daily **(A)**. Survival probability **(B)**. Body weight as percent of starting weight **(C)**. H&E **(D)**, and NP antigen-stained **(E)** representative lungs from animals euthanized at day 6 post-challenge. Data are representative of two independent experiments with n=10/group. Symbols represent group averages, bars represent SD **(C)**. *p<0.05, **p<0.01, ***p<0.001, ****p<0.0001 by Dunnett’s multiple comparison **(C)**, or Mantel-Cox Log-rank test **(B)**.

DBA/2J mice were immunized once with 10µg pVACC-NP^H3^ and 0.5μg of pIL-12, or 10μg of pHA^H1^ and 0.5μg of pIL-12, or co-immunized with a combination formulation of pHA^H1^, pVACC-NP^H3^, and pIL-12 (Combo) (**Figure 4A**). Animals were challenged fourteen days post-immunization with 100 LD_50_ Ca09-X179A. Animals which received pVACC-NP^H3^ and pIL-12 succumbed to this lethal challenge by day 7, as did naïve animals, however 100% of mice which received pHA^H1^ and pIL-12 or the combination vaccine survived challenge (**Figure 4B**). All pHA^H1^ and pIL-12 immunized animals lost significant weight but survived challenge (100%). Interestingly, only the Combo group afforded complete protection from both mortality (**Figure 4B**) and morbidity as measured by weight loss (**Figure 4C**). H&E staining revealed dense cellular infiltrates in the lungs of naïve and pVACC-NP^H3^-only immunized mice (**Figure 4D**). pHA^H1^-only and combination-immunized mouse lungs displayed more open airway space but had intermediate cellular infiltration and modest evidence of alveolar wall thickening (**Figure 4D**). When sections were stained for NP antigen, naïve animals had significant, dispersed NP antigen staining (**Figure 4E**). pVACC-NP^H3^-only immunized mouse lungs exhibited dense NP antigen staining which was localized to the alveolar spaces. In pHA^H1^-only immunized mouse lungs, NP staining was faint and dispersed, whereas Combination-immunized lungs display minimal NP positivity (**Figure 4E**). Taken together, these data support that the combination delivery of NP with HA antigens can improve challenge outcomes.

### 3.6 Inhibitory gene expression signatures are detected in lungs during infection in mice receiving pHA^H1^ or pVACC-NP^H3^ DNA vaccines

**Figure 5:**
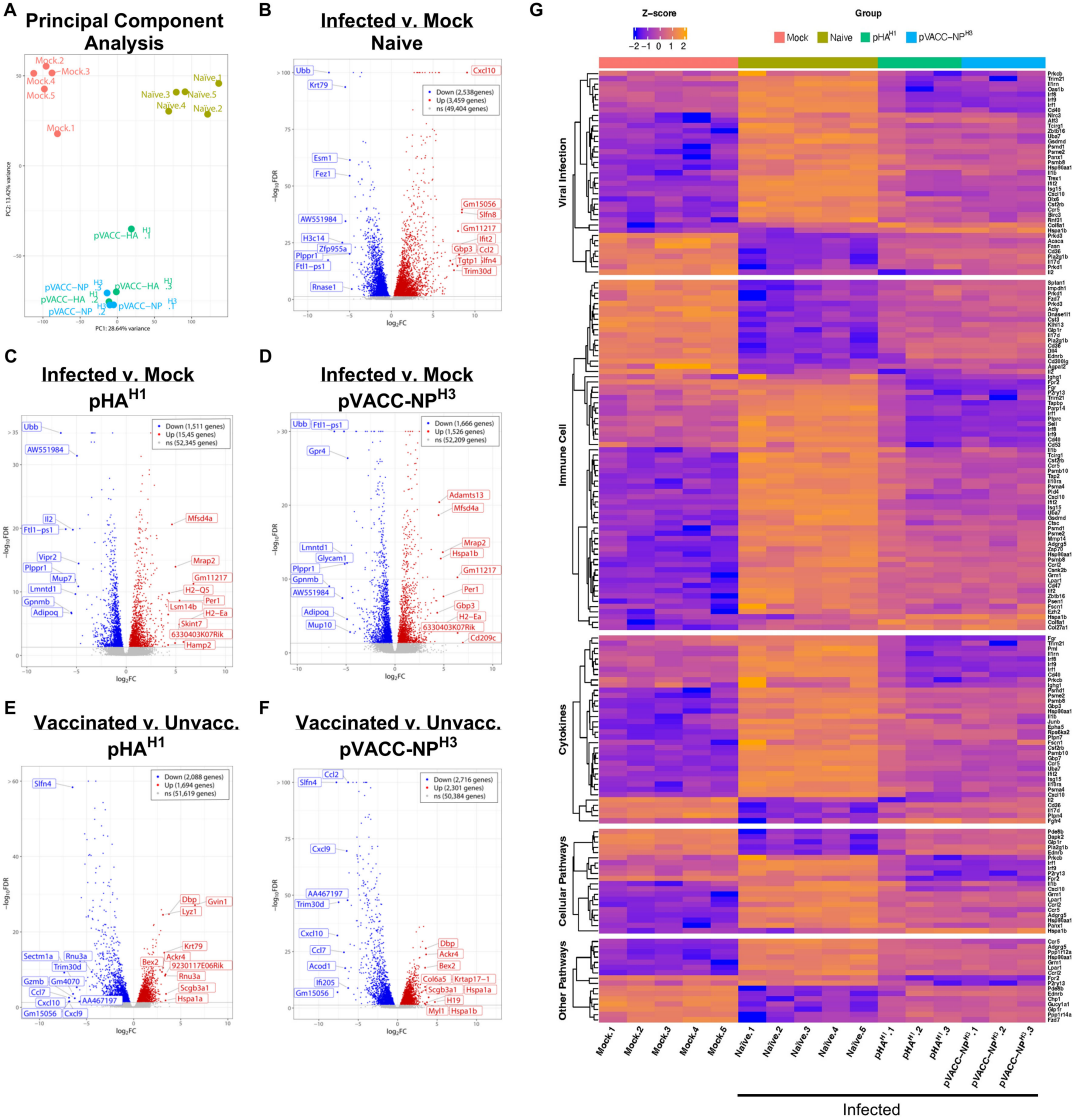
Differential gene expression (DEG) analysis of pHA^H1^ and pVACC-NP^H3^ DNA vaccines during infection. Mice (DBA/2J) were immunized with one dose of pHA^H1^ or pVACC-NP^H3^ and 14 days later, challenged with 10 LD50 of Ca09-X179A virus. Total RNA was extracted from FFPE scrolls of lung harvested at day-6 post-infection. Control groups included mock (unvaccinated, uninfected) and naïve (unvaccinated, infected). **(A)** PCA analysis of all four groups. Volcano plots comparing infection versus mock are shown for **(B)** naïve, **(C)** pHA^H1^, and **(D)** pVACC-NP^H3^. Volcano plots comparing vaccinated versus unvaccinated mice are shown for **(E)** pHA^H1^, and **(F)** pVACC-NP^H3^. The top 10 upregulated and 10 downregulated genes are highlighted for all volcano plots. **(G)** Ingenuity pathway analysis comparing significant gene signatures associated with viral infection, immune cells, cytokines, and other pathways across all groups. Data representing pHA^H1^ or pVACC-NP^H^ are from one independent experiment with n=3/group. Data representing naïve and mock are from 2 independent experiments with n=3 and n=2 per group (total n=5/group).

To evaluate the impact of DNA vaccination on host transcription signatures, differential gene expression (DEG) in naïve, pHA^H1^, and pVACC-NP^H3^ vaccinated mice were profiled by 3’mRNA-Seq following infection with 10 LD_50_ Ca09-X179A. A mock (uninfected) group receiving PBS alone was run in parallel and included as control. Principal component analysis indicated distinct clustering of the mock, naïve, and vaccinated groups, with both pHA^H1^ and pVACC-NP^H3^ co-localizing (**Figure 5A**). We compared DEGs between mock animals and those that were either naïve (unvaccinated) (**Figure 5B**), immunized with pHA^H1^ (**Figure 5C**), or immunized with pVACC-NP^H3^ (**Figure 5D**). Compared with mock, DEG analysis of the naïve group found 2, 538 genes downregulated (blue) and 3, 459 genes upregulated (red). pHA^H1^ analysis found 1, 511 genes downregulated and 1, 545 genes upregulated. Finally, DEG analysis of pVACC-NP^H3^ indicated 1, 666 genes down and 1, 525 genes upregulated. The top 20 DEGs in total are displayed on each of the volcano plots. We next compared DEG signatures for pHA^H1^ (**Figure 5E**) and pVACC-NP^H3^ (**Figure 5F**) vaccinated mice with the naïve (unvaccinated) group. DEG analysis for pHA^H1^ found 2, 088 downregulated genes and 1, 694 upregulated genes. Analysis for pVACC-NP^H3^ found 2, 716 genes downregulated and 2, 301 genes upregulated. Again, the top 20 up and downregulated genes in total are highlighted for each comparison. Influenza infection was associated with significant increases in virus-associated and inflammatory gene signatures, including CXC-motif chemokine ligand-10 or interferon gamma induced protein-10 (CXCL10/IP-10), a well-characterized influenza infection-induced inflammatory mediator (53–56). Importantly, immunization with either pHA^H1^ or pVACC-NP^H3^ significantly decreased CXCL-10 gene expression. For pVACC-NP^H3^ vaccinated animals this decrease was statistically significant, and we observed statistically significant increases in other inflammatory mediators including CCL2 (57), CXCL-9 (54), and CCL7 (58). Together with our challenge data, these results suggest that DNA immunization with influenza antigens supports decreased influenza-associated inflammation.

Pathway analysis identified the activation of pathways associated with viral infection, immune cells, cytokines, cellular pathways, and other pathways naïve (unvaccinated, infected) group, compared with the mock (unvaccinated, uninfected) control animals (**Figure 5G**, **Supplementary Figure S7**). Very few significant differences in DEG signatures based on p-value were observed between pHA^H1^ or pVACC-NP^H3^ vaccinated animals compared with the mock group and all had a false discovery rate (FDR) >10% and low significance (**Supplementary Figures S7B, C**). Analysis of pHA^H1^ or pVACC-NP^H3^ vaccinated signatures found the converse, where pathways associated with viral infection, immune cells, cytokines, and cellular pathways were overall inhibited (**Figure 5G**, **Supplementary Figures S8**, **S9**). Overall, these data highlight the protective impact of pHA^H1^ or pVACC-NP^H3^ priming to control genes associated with response to IAV infection.

### 3.7 DNA-encoded VACC-NP^X^ antigens induce durable T cell responses in mice

To evaluate the longevity of pVACC-NP^X^-induced cellular responses, C57BL/6J mice were immunized twice, separated by three weeks with pVACC-NP^H1^ alone, pVACC-NP^H3^ alone, or co-immunized with each immunogen and plasmid-encoded IL-12 (+pIL-12) and rested for ~200 days (6 months) (**Supplementary Figure S10A**). T cell responses were detected by IFNγ ELISPOT assay with the spleens and lungs of mice following immunization. At this memory timepoint, significant anti-NP responses in the spleen and lungs for both pVACC-NP^H1^ (**Supplementary Figures S10B, D**, respectively) and pVACC-NP^H3^ (**Supplementary Figure S6C, E**, respectively) compared to naïve controls. Co-delivery of pIL-12 demonstrated long-term enhancement of IFNγ secretion in both compartments compared with animals receiving pVACC-NP^H1^ (**Supplementary Figures S10B, D**, respectively) and pVACC-NP^H3^ (**Supplementary Figures S10C, E**, respectively). These data indicate that VACC-NP^X^ DNA vaccines can elicit robust and long-lived cellular responses *in vivo* and highlight the potent contribution of molecular adjuvant pIL-12 to enhancing cellular immunity at acute and memory timepoints post-vaccination.

## 4 Discussion

Conventional seasonal influenza vaccines induce antibodies primarily directed against the surface HA glycoprotein. This protection is most optimal against matched and minimally mutated strains, with hemagglutination inhibition (HAI) titers >1:40 associated with protection in 50% of people (59, 60). Even moderate antigenic drift can dramatically reduce vaccine effectiveness of traditional inactivated and LAIV vaccines (61, 62). In parallel, cellular immune responses inducing both CD4^+^ and CD8^+^ T cell responses can provide important early protection (63, 64) and IAV clearance [reviewed in (65) and (66)] including correlating with decreased recovery time (67). Therefore, directing immune responses to highly conserved internal antigens could be a viable approach to supplement anti-HA directed antibodies with anti-IAV targeting cellular responses. Of these internal antigens, the conserved IAV NP is an attractive target for broad and universal influenza strategies (16–19) and, in humans, anti-NP cytotoxic T lymphocyte responses can reduce pathogenesis and confer important heterosubtypic protection (68). Here, we describe the design and immunogenicity of two new synthetic NP immunogens guided by the genetic sequences obtained from the seasonal IAV-H1N1 and IAV-H3N2 WHO-recommended vaccine strains. Our data demonstrates the ability of single NP DNA immunization to rapidly to protect against mortality in a Ca09-X179A challenge model and further shows additive activity in combination with a pHA^H1^ DNA vaccine, achieving full protection when administered only two weeks before lethal challenge.

Synthetic NP immunogens have been evaluated in various platforms including DNA (69–71), mRNA (72, 73), and viral vector-based platforms such as adenovirus (74) and modified vaccinia Ankara (MVA) (75, 76) vectors. In addition to strain-matched designs, approaches to targeting IAV-NP include CD8^+^ T cell epitope-based strains and oligomerized forms (77, 78). Here, we designed a synthetic consensus immunogen with the goal of inducing broadly protective cellular responses. This vaccine-aligned common consensus approach, or VACC, leverages our synthetic-consensus (SynCon) approach (79–83) to generate single NP immunogens representing conserved features of the IAV-NP^H1^ and proteins. The number of sequenced circulating influenza strains has dramatically increased with advancements in sequencing technologies and one approach for immunogen design is to computationally align thousands of IAV NP sequences to generate a single sequence (84). Alternatively, the pVACC approach focuses specifically on vaccine strains; Since the 1970s, the WHO has provided recommendations for the composition of seasonal influenza vaccines. This requires yearly surveillance involving analysis of clinical specimens, disease burden, and epidemiological data to understand representative viruses in the human population and their distribution by country and region (85). The selected vaccine viruses could therefore be considered as representative of the diversity of major influenza viruses circulating in the human population in a current year. Using this as a guide, the VACC-NP^X^ candidates therefore encompass yearly NP variation. Although fewer sequences are aligned, the VACC approach reduces bias from sequence variability and quality. Supporting our approach, vaccination with these *de novo* DNA immunogens induced strong T cell responses in the spleen and lungs and protected against lethal Ca09-X179A challenge in mice.

Both pVACC-NP^H1^ and pVACC-NP^H3^ elicited robust T cell responses that were durable in mice and our data show the protective potency of targeting the IAV-NP, achieving single dose protection 14 days following delivery in mice. Interestingly, although both VACC-NP constructs protected against death, we observed interesting superior prevention of IAV-associated pathogenesis in the lungs of DBA/2J mice immunized with the heterologous pVACC-NP^H3^ DNA vaccine. In our studies, the Ca09-X179A challenge virus contains the internal proteins including NP from A/Puerto Rico/8/1934 (PR8). The H1N1pdm09 triple reassortant event, resulted in introduction of a classical swine NP, resulting in the PR8-NP being 8.7% different in sequence to Ca09. Interestingly, the synthetic VACC-NP^H3^ design is almost equidistant, with 9.0% different from the PR8-NP and 10.7% from Ca09, respectively (**Supplementary Figure S11**). Further studies with mouse-adapted IAV and different mouse strains could provide additional input into this heterologous protection. pVACC-NP^H3^ displayed better *in vivo* immunogenicity in both spleen and lungs. In other work, we have demonstrated the importance of nucleotide and amino acid changes towards *in vivo* expression (86, 87) and it is possible that this could contribute to the difference in immunogenicity observed between these constructs.

While NP generates a robust cytotoxic T lymphocyte (CTL) response and can potentially contribute to humoral immunity (88), it remains likely that an HA immunogen component will be essential in IAV vaccine formulations to provide robust antibody-mediated protection. We evaluated the inclusion of pIL-12 in NP-only and HA+NP formulations in these studies. Addition of pIL-12 to NP-only formulations led to trends toward increased NP-specific T cell responses. This pattern of increased NP-specific T cell responses was also observed when pIL-12 was added to the combination of pVACC-NP^H3^ and pHA^H1^. However, for HA-specific T cell responses, only groups receiving pHA^H1^ generated HA-specific cellular responses. The generation of T cell responses against both HA and NP peptides highlights the potential for both antigens to be co-administered, at least in mice. Importantly, mice immunized with a suboptimal dose of pHA^H1^ or pVACC-NP^H3^ alone did not achieve complete protection. However, pHA^H1^ plus pVACC-NP^H3^ co-immunized animals were completely protected from morbidity and mortality. These data indicate that pVACC-NP^H3^, and indeed other NP immunogens, can play a role to complement HA-based vaccine-induced immunity. Additional studies dissecting this synergy, likely due to T cell immunity, would be interesting and evaluation in mice and larger models would be informative for dose titration and combination studies. Interestingly, we observed lower T cell responses when both pHA^H1^ plus pVACC-NP^H3^ were co-delivered, consistent with potential interference which could impede immune responses. Although protective in the DBA/2J mouse model, further studies dissecting immune responses associated with HA and NP antigen combination will be important to understanding the impact of this decreased immunogenicity.

One possible limitation of our approach is the focus on human seasonal IAV-H1 and IAV-H3. As highlighted, the current circulating human IAV-H3 viruses have varied minimally over the past 20 years (<1.1%). In 2009, the introduction of the reassortant A/H1N1pdm09 swine flu viruses into the human population resulted in the introduction of a classical swine H1N1 NP into humans (45, 46), a significant antigenic shift (>10%). We therefore focused the VACC-NP^H1^ design based on post-H1N1pdm09 viruses. To further address major antigenic shift events, additional consideration of animal (for example swine) H1 and H3 circulating strains would be valuable. Although there is no global body selecting vaccine strains for animals, similar surveillance of strains circulating in animals is being undertaken by various agencies such as the United States and European Centers for Disease Control (CDC) and others, alerting to emerging influenza strains with potential for zoonotic crossover into humans. Yearly monitoring and selection of predominantly circulating animal IAV would be valuable for narrowing down and selecting strains for inclusion in immunogen design. Such animal IAV-H1 and IAV-H3 NP immunogens could be incorporated as multivalent combinations to elicit broader cellular immune responses against potential emerging viruses.

It should be emphasized, that our study demonstrates the potency of pVACC candidates to rapidly induce protective immune responses with a single immunization, achieving protection against a 10 LD50 challenge within only 14 days with immunogens designed to elicit primarily T cell responses. These data are further supported by transcriptomic analysis identifying genes and pathways associated with inhibitory control of viral infection and inflammation in pHA^H1^ or pVACC-NP^H3^ vaccinated mice. These include inhibition of genes associated with innate immune cell activation, cross-talk, and lymphocyte activation and indicative of vaccine-related immune priming to control infection. Further, we show that the NP^H3^ immunogen can adjuvant the pHA^H1^ vaccine in a more stringent 100 LD50 challenge model. Our approach to generating synthetically designed NP antigens based on yearly vaccine strains can be broadly applied to other highly conserved influenza internal proteins with potential to generate robust protective CTL responses. Additional studies evaluating protective efficacy in H3N2 challenges and other heterologous subtypes would be valuable. Further studies evaluating protective efficacy at later time points following maturation of the immune response and the inclusion of prime-boost regimens will also be valuable. The NP antigen co-delivery has potential to enhance various vaccine platofmrs and further study with commercially available, seasonal HA-based vaccine regimens and other delivery platforms would be insightful. In summary, these data support the incorporation of the VACC design approach for continued development of broad and efficacious influenza interventions.

## Data Availability

The data presented in the study are deposited in the NCBI GEO repository, accession number GSE306862.

## Supplementary material

Supplementary Table 1 Influenza NP peptides and pools for ICS and ELISpot assays. **(A)** pVACC-NPH1 peptides and pools. **(B)** pVACC-NPH3 peptides and pools. **(C)** Peptide matrix pool design schematic.

Supplementary Table 2 Ingenuity Pathway analysis of naïve versus mock mice.

Supplementary Table 3 Ingenuity Pathway analysis of pHA^H1^ versus mock mice.

Supplementary Table 4 Ingenuity Pathway analysis of pVACC-NP^H3^ versus mock mice.

Supplementary Table 5 Ingenuity Pathway analysis of pHA^H1^ versus naïve mice.

Supplementary Table 6 Ingenuity Pathway analysis of pVACC-NP^H3^ versus naïve mice.

Supplementary Figure 1 **(A)**The H1N1 and H3N2 vaccine strains included in the generation of the vaccine pVACC-NP^H1^ and pVACC-NP^H3^. **(B)** A phylogenetic tree

Supplementary Figure 2 Multiple sequence alignment of NP proteins of seasonal A/H1N1 vaccine strains, performed in Geneious Prime. Dots represent complete identity of the residue in all the sequences. The green bar on top represents the level of conservation of the residues between strains, deep green indicates most conserved, yellow indicates a poorly conserved position.

Supplementary Figure 3 Multiple sequence alignment of NP proteins of seasonal A/H3N2 vaccine strains, performed in Geneious Prime. Dots represent complete identity of the residue in all the sequences. The green bar on top represents the level of conservation of the residues between strains, deep green indicates most conserved, yellow indicates a poorly conserved position.

Supplementary Figure 4 A 2-injection regimen of VACC-NP^H3^ induces robust immune responses in C57BL/6J mice. C57BL/6J mice were immunized with 10µg or 25µg of pVACC-NP^H3^ plasmid at a 21-day interval and euthanized seven days post-immunization for cellular analyses **(A)**. IFNγ spot-forming units (SFUs) in spleens following stimulation with H3NP peptides (n=5 mice per group) **(B)**.

Supplementary Figure 5 Identification of pVACC-NP^X^ immunodominant T cell epitopes. **(A)** C57BL/6J mice were immunized twice, separated by three weeks with 10μg of pVACC-NP^X^. Matrix peptide pools were used to stimulate isolated splenocytes. **(B)** H1N1-NP specific IFNγ secretion as measured by ELISpot. **(C)** Identified H1N1-NP immunodominant peptides. **(D)** H3N2-NP specific IFNγ secretion as measured by ELISpot. **(E)** Identified H3N2-NP immunodominant peptides. Data are representative of one experiment with n=5/group. Symbols represent the average of duplicate assays per animal, bars represent group mean, error bars represent SEM.

Supplementary Figure 6 pHA^H1^ is sub-protective in high-dose IAV challenge: **(A)** Immunofluorescence staining of HEK293T cells transfected with pHA_H1_ plasmid and stained for IAV-NP. **(B)** Mice were immunized once with 10μg, 1μg, or 0.5μg of plasmid-encoded A/California/07/2009 HA (pHA^H1^) and challenged with 100 LD50 of Ca09-X179A virus fourteen days later. **(C)** Survival probability. **(D)** Body weights as percent of starting weight. Data are representative of one experiment with n=10/group. Symbols represent group mean, error bars represent SD. ***p<0.001, ****p<0.0001 Mantel-Cox Log-rank test **(C)** ns = not significant by Dunnett’s multiple comparison test **(D)**.

Supplementary Figure 7 VACC-NP^X^ immunogens induce robust and durable cellular responses *in vivo*. **(A)** Female C57BL/6J mice were immunized twice separated by three weeks and mice were rested for 200 days (six months) with 10 μg of pVACC-NP^H1^ or pVACC-NP^H3^ alone, or co-immunized with pVACC-NP^x^ and 0.5μg of plasmid-encoded IL-12 (+pIL-12). **(B)** NP^H1^-specific IFNγ spot-forming units (SFU) in spleens and **(D)** lungs. **(C)** NP^H3^-specific IFNγ spot-forming units (SFU) in spleens **(E)** and lungs. Data are representative of one experiment with n=5/group. Bars represent the mean; error bars represent SEM (C-J) or SD (K-R). **p<0.01, ***p<0.001, ****p<0.0001 by Two-way ANOVA.

Supplementary Figure 8 Ingenuity pathway analysis (IPA) of versus Mock (unvaccinated, uninfected). IPA was performed on the total gene expression dataset and further categorized into subsets involved with viral infection, immune cells, cytokines, cellular pathways, and other pathways. Bar charts comparing the number of activated and inhibited pathways are shown for **(A)** naïve versus mock, **(B)** pHA^H1^ versus mock, and **(C)** pVACC-NP^H3^ versus mock groups. Groups were first filtered by Z-score >2.0 and a false discovery rate of 0.05. All groups have significant p-values <0.05.

Supplementary Figure 9 Ingenuity pathway analysis (IPA) of pHA^H1^ versus Naïve. IPA was performed on the total gene expression dataset and further categorized into subsets involved with viral infection, immune cells, cytokines, cellular pathways, and other pathways. Bar charts comparing the number of activated and inhibited pathways are shown for pHA^H1^ versus naïve. Groups were first filtered by Z-score >2.0 and a false discovery rate of 0.05.

Supplementary Figure 10 Ingenuity pathway analysis (IPA) of pVACC-NP^H3^ versus Naïve. IPA was performed on the total gene expression dataset and further categorized into subsets involved with viral infection, immune cells, cytokines, cellular pathways, and other pathways. Bar charts comparing the number of activated and inhibited pathways are shown for pVACC-NP^H3^ versus naïve. Groups were first filtered by Z-score >2.0 and a false discovery rate of 0.05.

Supplementary Figure 11 Phylogenetic analysis comparing pVACC-NP^X^ immunogens with A/California/07/2009 and Ca09-X179A (PR8) NP proteins.
