# Supplementary material for: Synthetic DNA co-immunization with vaccine-aligned common consensus nucleoprotein and hemagglutinin protects mice against lethal influenza infection with a single immunization
Source: Front Immunol. 2025 Nov 26;16:1632121. doi: 10.3389/fimmu.2025.1632121 (PMC12689538; doi:10.3389/fimmu.2025.1632121)
Supplement: Supplementary Table 2 — Ingenuity Pathway analysis of naïve versus mock mice. [file DataSheet2.pdf]

Pathways	P	FDR	State	Z	N	Pos	Neg	Molecules
Pathogen Induced Cytokine Storm Signaling Pathway	2.512E-17	0%	Activated	5.812	76	64	12	[illegible]
Cell Cycle Checkpoints	3.311E-05	0%	Activated	5.284	39	36	3	[illegible]
Interferon alpha/beta signaling	1.585E-13	0%	Activated	5.196	27	27	0	[illegible]
Interferon gamma signaling	3.09E-10	0%	Activated	5.196	27	27	0	[illegible]
Role of Hypercytokinemia/hyperchemokinemia in the Pathogenesis of Influenza	1.072E-09	0%	Activated	4.6	25	24	1	[illegible]
Interleukin-10 signaling	7.943E-11	0%	Activated	4.359	19	19	0	[illegible]
S100 Family Signaling Pathway	2.692E-06	0%	Activated	4.298	90	65	25	[illegible]
Deubiquitination	0.0154882	8%	Activated	4.271	29	26	3	[illegible]
ISGylation Signaling Pathway	0.0007244	1%	Activated	4.243	18	18	0	[illegible]
TCR signaling	0.0041687	3%	Activated	4.243	18	18	0	[illegible]
TREM1 Signaling	1.698E-06	0%	Activated	4.123	18	18	0	[illegible]
Mitotic G2-G2/M phases	0.0095499	6%	Activated	4.082	24	22	2	[illegible]
Immunoregulatory interactions between a Lymphoid and a non-Lymphoid cell	0.0204174	9%	Activated	3.962	23	21	2	[illegible]
Phagosome Formation	1.514E-05	0%	Activated	3.889	81	58	23	[illegible]
Neutrophil degranulation	5.623E-07	0%	Activated	3.845	65	48	17	[illegible]
Neuroinflammation Signaling Pathway	1.096E-05	0%	Activated	3.795	44	34	10	[illegible]
Role of Macrophages, Fibroblasts and Endothelial Cells in Rheumatoid Arthritis	7.079E-07	0%	Activated	3.753	49	37	12	[illegible]
Costimulation by the CD28 family	0.0012882	1%	Activated	3.742	14	14	0	[illegible]
Multiple Sclerosis Signaling Pathway	2.344E-10	0%	Activated	3.727	45	38	7	[illegible]
Macrophage Classical Activation Signaling Pathway	7.586E-08	0%	Activated	3.667	36	32	4	[illegible]
Protein Ubiquitination Pathway	0.0147911	7%	Activated	3.657	30	26	4	[illegible]
Class A/1 (Rhodopsin-like receptors)	2.399E-05	0%	Activated	3.618	44	34	10	[illegible]
TNFR2 non-canonical NF-kB pathway	0.0010471	1%	Activated	3.606	13	13	0	[illegible]
Breast Cancer Regulation by Stathmin1	3.802E-05	0%	Activated	3.586	70	48	22	[illegible]
IL-27 Signaling Pathway	2.754E-08	0%	Activated	3.528	29	26	3	[illegible]
Telomere Maintenance	0.0058884	4%	Activated	3.464	12	12	0	[illegible]
Mitotic Prometaphase	0.0064565	4%	Activated	3.4	25	21	4	[illegible]
BBSome Signaling Pathway	0.0034674	3%	Activated	3.394	52	40	12	[illegible]
Histone Modification Signaling Pathway	0.0085114	5%	Activated	3.307	33	26	7	[illegible]
Role of JAK family kinases in IL-6-type Cytokine Signaling	1.047E-05	0%	Activated	3.3	18	18	0	[illegible]
Pyroptosis Signaling Pathway	2.818E-06	0%	Activated	3.273	21	18	3	[illegible]
CGAS-STING Signaling Pathway	2.455E-06	0%	Activated	3.272	27	23	4	[illegible]
IL-17 Signaling	0.0004467	1%	Activated	3.272	27	21	6	[illegible]
CREB Signaling in Neurons	0.0001288	0%	Activated	3.25	68	46	22	[illegible]
Interferon Signaling	8.511E-08	0%	Activated	3.207	14	14	0	[illegible]
IL-17A Signaling in Fibroblasts	0.0016596	2%	Activated	3.207	14	13	1	[illegible]
Th1 Pathway	1.995E-11	0%	Activated	3.182	33	28	5	[illegible]
Antigen Presentation Pathway	0.0003467	0%	Activated	3.162	10	10	0	[illegible]
Tumor Microenvironment Pathway	4.898E-06	0%	Activated	3.157	31	27	4	[illegible]
Interleukin-4 and Interleukin-13 signaling	5.754E-08	0%	Activated	3.138	26	24	2	[illegible]
NOD1/2 Signaling Pathway	1.514E-05	0%	Activated	3.053	31	25	6	[illegible]
Cachexia Signaling Pathway	2.754E-06	0%	Activated	3.051	52	39	13	[illegible]
Interleukin-2 family signaling	1.514E-05	0%	Activated	3.051	13	12	1	[illegible]
Crosstalk between Dendritic Cells and Natural Killer Cells	2.291E-05	0%	Activated	3.051	19	17	2	[illegible]
Retinoic acid Mediated Apoptosis Signaling	7.413E-05	0%	Activated	3.051	14	12	2	[illegible]
HER-2 Signaling in Breast Cancer	0.0039811	3%	Activated	3.024	28	18	10	[illegible]
TP53 Regulates Transcription of Cell Cycle Genes	0.0077625	5%	Activated	3	9	9	0	[illegible]
Mitotic Metaphase and Anaphase	0.0338844	13%	Activated	3	25	20	5	[illegible]
Role of Pattern Recognition Receptors in Recognition of Bacteria and Viruses	5.012E-12	0%	Activated	2.985	39	33	6	[illegible]
Molecular Mechanisms of Cancer	1.585E-12	0%	Activated	2.921	119	80	39	[illegible]
KEAP1-NFE2L2 pathway	0.030903	12%	Activated	2.887	12	11	1	[illegible]
Xenobiotic Metabolism PXR Signaling Pathway	0.0038019	3%	Inhibited	-2.858	25	4	21	[illegible]
FXR/RXR Activation	0.0044668	3%	Inhibited	-2.858	24	13	11	[illegible]
Role of PKR in Interferon Induction and Antiviral Response	3.981E-05	0%	Activated	2.837	24	22	2	[illegible]
Cytosolic sensors of pathogen-associated DNA	0.0162181	8%	Activated	2.828	8	8	0	[illegible]
Meiotic recombination	0.020893	9%	Activated	2.828	8	8	0	[illegible]
Nucleosome assembly	0.0234423	10%	Activated	2.828	8	8	0	[illegible]
Cellular Effects of Sildenafil (Viagra)	0.0004074	1%	Inhibited	-2.79	73	49	24	[illegible]
Antioxidant Action of Vitamin C	0.0079433	5%	Inhibited	-2.714	16	12	4	[illegible]
DDX58/IFIH1-mediated induction of interferon-alpha/beta	0.0263027	11%	Activated	2.714	11	10	1	[illegible]
RHOGDI Signaling	0.047863	17%	Activated	2.714	23	5	18	[illegible]
Ribonucleotide Reductase Signaling Pathway	0.0029512	2%	Activated	2.711	23	19	4	[illegible]
PPAR Signaling	0.0042658	3%	Inhibited	-2.673	16	14	2	[illegible]
Oxidative Phosphorylation	0.0316228	13%	Inhibited	-2.673	14	2	12	[illegible]
Immunogenic Cell Death Signaling Pathway	0.0002239	0%	Activated	2.668	17	14	3	[illegible]
GPVI-mediated activation cascade	0.0114815	6%	Activated	2.646	7	7	0	[illegible]
G alpha (i) signalling events	0.020893	9%	Activated	2.646	28	21	7	[illegible]
Atherosclerosis Signaling	9.12E-06	0%	Activated	2.558	25	20	5	[illegible]
CDX Gastrointestinal Cancer Signaling Pathway	0.0114815	6%	Inhibited	-2.558	24	17	7	[illegible]
Xenobiotic Metabolism CAR Signaling Pathway	0.0131826	7%	Inhibited	-2.558	23	3	20	[illegible]
Interleukin-3, Interleukin-5 and GM-CSF signaling	0.0056234	4%	Activated	2.53	10	9	1	[illegible]
Cell Cycle Control of Chromosomal Replication	0.0063096	4%	Activated	2.53	10	9	1	[illegible]
LPS/IL-1 Mediated Inhibition of RXR Function	0.0066069	4%	Activated	2.53	30	7	23	[illegible]
Metabolism of water-soluble vitamins and cofactors	0.0093325	5%	Inhibited	-2.53	10	1	9	[illegible]
Pancreatic Adenocarcinoma Signaling	0.0194984	9%	Activated	2.53	16	13	3	[illegible]
NAD Signaling Pathway	0.0128825	7%	Activated	2.524	19	15	4	[illegible]
Kinetochore Metaphase Signaling Pathway	0.0023442	2%	Activated	2.496	17	16	1	[illegible]
Wound Healing Signaling Pathway	0.0013804	1%	Activated	2.475	32	23	9	[illegible]
Role of IL-17F in Allergic Inflammatory Airway Diseases	1.95E-05	0%	Activated	2.449	13	13	0	[illegible]
UVA-Induced MAPK Signaling	0.0017378	2%	Activated	2.449	16	15	1	[illegible]
Differential Regulation of Cytokine Production in Intestinal Epithelial Cells by IL-17A and IL-17F	0.0048978	4%	Activated	2.449	6	6	0	[illegible]
Interleukin-6 family signaling	0.006166	4%	Activated	2.449	6	6	0	[illegible]
Activin Inhibin Signaling Pathway	0.0186209	9%	Activated	2.449	24	19	5	[illegible]
IL-33 Signaling Pathway	0.0077625	5%	Activated	2.4	23	19	4	[illegible]
RAR Activation	0.0003311	0%	Inhibited	-2.38	51	32	19	[illegible]
Interleukin-1 family signaling	0.0053703	4%	Activated	2.357	18	14	4	[illegible]
Role of Osteoclasts in Rheumatoid Arthritis Signaling Pathway	2.57E-05	0%	Activated	2.343	41	28	13	[illegible]
Guanosine Nucleotides Degradation III	0.0001549	0%	Activated	2.236	6	6	0	[illegible]
Adenosine Nucleotides Degradation II	0.0006026	1%	Activated	2.236	6	6	0	[illegible]
iNOS Signaling	0.0058884	4%	Activated	2.236	8	8	0	[illegible]
Differential Regulation of Cytokine Production in Macrophages and T Helper Cells by IL-17A and IL-17F	0.0075858	5%	Activated	2.236	5	5	0	[illegible]
Interleukin-12 family signaling	0.0123027	6%	Activated	2.236	5	5	0	[illegible]
Inflammasome pathway	0.0123027	6%	Activated	2.236	5	5	0	[illegible]
Ethanol Degradation IV	0.030903	12%	Inhibited	-2.236	5	0	5	[illegible]
CSDE1 Signaling Pathway	0.0467735	17%	Activated	2.236	8	7	1	[illegible]
Role of Chondrocytes in Rheumatoid Arthritis Signaling Pathway	2.57E-05	0%	Activated	2.2	25	19	6	[illegible]
NAFLD Signaling Pathway	1.585E-05	0%	Activated	2.197	35	24	11	[illegible]
Hematoma Resolution Signaling Pathway	0.0002455	0%	Inhibited	-2.197	35	22	13	[illegible]
3-phosphoinositide Biosynthesis	0.0489779	17%	Activated	2.183	22	16	6	[illegible]
Xenobiotic Metabolism AHR Signaling Pathway	0.0004571	1%	Inhibited	-2.138	16	5	11	[illegible]
Role of MAPK Signaling in Inhibiting the Pathogenesis of Influenza	0.0014791	1%	Activated	2.138	14	12	2	[illegible]
PD-1, PD-L1 cancer immunotherapy pathway	1.95E-06	0%	Inhibited	-2.132	23	21	2	[illegible]
Glutathione-mediated Detoxification	0.0012589	1%	Inhibited	-2.121	8	1	7	[illegible]
Lymphotoxin β Receptor Signaling	0.0056234	4%	Activated	2.121	10	9	1	[illegible]
Autism Signaling Pathway	0.0154882	8%	Activated	2.121	33	22	11	[illegible]
Th2 Pathway	1.585E-06	0%	Activated	2.117	27	24	3	[illegible]
Colorectal Cancer Metastasis Signaling	0.0012303	1%	Activated	2.117	34	23	11	[illegible]
NLR signaling pathways	0.0019953	2%	Activated	2.111	11	9	2	[illegible]
Role of Osteoblasts in Rheumatoid Arthritis Signaling Pathway	0.0001778	0%	Activated	2.058	34	23	11	[illegible]
Cardiac Hypertrophy Signaling (Enhanced)	1.202E-05	0%	Activated	2.023	66	41	25	[illegible]
Airway Pathology in Chronic Obstructive Pulmonary Disease	0.0020417	2%		2	18	14	4	[illegible]
OAS antiviral response	0.0025704	2%		2	4	4	0	[illegible]
Pathogenesis of Multiple Sclerosis	0.0025704	2%		2	4	4	0	[illegible]
nNOS Signaling in Neurons	0.0186209	9%		-2	7	1	6	[illegible]
FAT10 Signaling Pathway	0.0204174	9%		2	9	9	0	[illegible]
HOTAIR Regulatory Pathway	0.0269153	11%		2	19	14	5	[illegible]
Airway Inflammation in Asthma	0.0288403	12%		2	6	5	1	[illegible]
Eicosanoid Signaling	0.0069183	4%		1.976	32	21	11	[illegible]
Activation of IRF by Cytosolic Pattern Recognition Receptors	4.786E-05	0%		1.941	15	15	0	[illegible]
Purine Nucleotides Degradation II (Aerobic)	2.692E-05	0%		1.89	8	7	1	[illegible]
Natural Killer Cell Signaling	9.333E-05	0%		1.89	29	26	3	[illegible]
Signaling by SCF-KIT	0.0331131	13%		1.89	7	6	1	[illegible]
RAF/MAP kinase cascade	0.0085114	5%		1.826	30	20	10	[illegible]
D-myo-inositol (1,4,5,6)-Tetrakisphosphate Biosynthesis	0.042658	16%		1.807	20	14	6	[illegible]
D-myo-inositol (3,4,5,6)-tetrakisphosphate Biosynthesis	0.042658	16%		1.807	20	14	6	[illegible]
Signaling by Rho Family GTPases	0.0489779	17%		-1.807	27	10	17	[illegible]
Cohesin Chromatin Regulation Pathway	0.0123027	6%		1.732	28	21	7	[illegible]
EPH-Ephrin signaling	0.0380189	15%		-1.732	12	3	9	[illegible]
p38 MAPK Signaling	0.001	1%		1.698	19	14	5	[illegible]
Macrophage Alternative Activation Signaling Pathway	8.71E-08	0%		1.667	36	27	9	[illegible]
Pyridoxal 5'-phosphate Salvage Pathway	0.0177828	9%		1.667	10	8	2	[illegible]
G-Protein Coupled Receptor Signaling	6.918E-06	0%		1.646	82	54	28	[illegible]
IL-23 Signaling Pathway	0.0051286	4%		1.633	9	9	0	[illegible]
FAT10 Cancer Signaling Pathway	0.0089125	5%		1.633	9	8	1	[illegible]
Pyroptosis	0.0112202	6%		1.633	6	5	1	[illegible]
Role of PI3K/AKT Signaling in the Pathogenesis of Influenza	0.0194984	9%		1.633	10	9	1	[illegible]
RIPK1-mediated regulated necrosis	0.0251189	11%		1.633	6	5	1	[illegible]
Activation of Matrix Metalloproteinases	0.0288403	12%		1.633	6	5	1	[illegible]
Gene and protein expression by JAK-STAT signaling after IL-12 stimulation	0.047863	17%		1.633	6	5	1	[illegible]
Parkinson's Signaling Pathway	0.0008511	1%		1.622	37	18	19	[illegible]
Leukocyte Extravasation Signaling	0.0033113	3%		1.606	25	17	8	[illegible]
Production of Nitric Oxide and Reactive Oxygen Species in Macrophages	1.862E-05	0%		1.569	31	21	10	[illegible]
Necroptosis Signaling Pathway	0.0039811	3%		1.528	21	15	6	[illegible]
NF-κB Activation by Viruses	0.0012882	1%		1.508	14	11	3	[illegible]
VDR/RXR Activation	0.0037154	3%		1.508	13	6	7	[illegible]
RHO GTPases Activate Formins	0.0436516	16%		1.5	16	11	5	[illegible]
PIP3 activates AKT signaling	0.0457088	17%		1.5	16	11	5	[illegible]
Death Receptor Signaling	2.63E-09	0%		1.46	25	22	3	[illegible]
IL-6 Signaling	0.0009772	1%		1.414	20	17	3	[illegible]
Sleep REM Signaling Pathway	0.001	1%		-1.414	18	13	5	[illegible]
Small Cell Lung Cancer Signaling	0.0091201	5%		1.414	14	12	2	[illegible]
TP53 Regulates Transcription of Cell Death Genes	0.0125893	7%		1.414	8	6	2	[illegible]
Dopamine-DARPP32 Feedback in cAMP Signaling	0.0489779	17%		-1.414	19	9	10	[illegible]
HIF1α Signaling	0.0047863	3%		1.4	26	16	10	[illegible]
Type II Diabetes Mellitus Signaling	0.0147911	7%		1.387	19	14	5	[illegible]
Hepatic Cholestasis	2.884E-05	0%		1.372	33	23	10	[illegible]
HMGB1 Signaling	2.512E-05	0%		1.342	28	19	9	[illegible]
Urate Biosynthesis/Inosine 5'-phosphate Degradation	0.000257	0%		1.342	6	5	1	[illegible]
Signal regulatory protein family interactions	0.0043652	3%		1.342	5	4	1	[illegible]
RHO GTPases activate CIT	0.0097724	6%		-1.342	5	1	4	[illegible]
RHO GTPases Activate ROCKs	0.0097724	6%		-1.342	5	1	4	[illegible]
RHO GTPases activate PAKs	0.0151356	7%		-1.342	5	1	4	[illegible]
Histamine Degradation	0.0151356	7%		-1.342	5	1	4	[illegible]
Fatty Acid α-oxidation	0.0151356	7%		-1.342	5	1	4	[illegible]
Role of RIG1-like Receptors in Antiviral Innate Immunity	0.0162181	8%		1.342	8	8	0	[illegible]
Putrescine Degradation III	0.018197	9%		-1.342	5	1	4	[illegible]
Oxytocin Signaling Pathway	0.0354813	14%		-1.3	28	12	16	[illegible]
Hepatic Fibrosis Signaling Pathway	0.002884	2%		1.298	45	29	16	[illegible]
Pulmonary Healing Signaling Pathway	0.030903	12%		1.279	22	14	8	[illegible]
Adrenergic Receptor Signaling Pathway (Enhanced)	0.0323594	13%		-1.279	22	14	8	[illegible]
Apoptotic execution phase	0.0037154	3%		1.265	10	7	3	[illegible]
GADD45 Signaling	0.0104713	6%		-1.265	10	9	1	[illegible]
Erythropoietin Signaling Pathway	0.0003981	1%		-1.225	26	20	6	[illegible]
Acute Phase Response Signaling	0.0003802	1%		1.213	27	21	6	[illegible]
Coronavirus Pathogenesis Pathway	0.0034674	3%		-1.177	26	23	3	[illegible]
cAMP-mediated signaling	0.0067608	4%		1.177	27	18	9	[illegible]
Semaphorin interactions	0.0019055	2%		-1.155	12	4	8	[illegible]
Complement System	0.0010233	1%		1.134	9	8	1	[illegible]
Growth Hormone Signaling	0.0001413	0%		-1.069	15	9	6	[illegible]
Inhibition of ARE-Mediated mRNA Degradation Pathway	0.0269153	11%		1.069	19	14	5	[illegible]
Induction of Apoptosis by HIV1	9.55E-08	0%		1	18	16	2	[illegible]
LXR/RXR Activation	2.239E-05	0%		-1	23	13	10	[illegible]
PI3K/AKT Signaling	0.0001122	0%		1	30	23	7	[illegible]
RUNX1 and FOXP3 control the development of regulatory T lymphocytes (Tregs)	0.0039811	3%		1	4	3	1	[illegible]
DNA damage-induced 14-3-3σ Signaling	0.0051286	4%		1	9	8	1	[illegible]
Synaptic Long Term Potentiation	0.0288403	12%		-1	15	7	8	[illegible]
RANK Signaling in Osteoclasts	0.030903	12%		1	11	9	2	[illegible]
LPS-stimulated MAPK Signaling	0.042658	16%		1	11	8	3	[illegible]
MIF-mediated Glucocorticoid Regulation	0.042658	16%		1	6	5	1	[illegible]
Angiopoietin Signaling	0.0467735	17%		-1	10	7	3	[illegible]
Degradation of the extracellular matrix	0.047863	17%		1	9	6	3	[illegible]
Ephrin A Signaling	0.0114815	6%		0.943	18	12	6	[illegible]
Role of BRCA1 in DNA Damage Response	0.0005495	1%		0.905	15	14	1	[illegible]
fMLP Signaling in Neutrophils	0.0275423	12%		-0.905	15	8	7	[illegible]
ATM Signaling	0.0281838	12%		0.905	13	10	3	[illegible]
G alpha (q) signalling events	0.0269153	11%		0.894	20	12	8	[illegible]
Neutrophil Extracellular Trap Signaling Pathway	0.000389	1%		0.866	48	31	17	[illegible]
Gαs Signaling	0.0017783	2%		0.832	19	12	7	[illegible]
ABC-family proteins mediated transport	0.0301995	12%		0.832	13	8	5	[illegible]
Fc Epsilon RI Signaling	0.0457088	17%		0.832	14	9	5	[illegible]
Docosahexaenoic Acid (DHA) Signaling	2.57E-05	0%		-0.822	37	23	14	[illegible]
Tumoricidal Function of Hepatic Natural Killer Cells	0.0011749	1%		0.816	7	6	1	[illegible]
IL-9 Signaling	0.0029512	2%		0.816	8	7	1	[illegible]
Growth hormone receptor signaling	0.006166	4%		0.816	6	4	2	[illegible]
Estrogen-mediated S-phase Entry	0.0093325	5%		0.816	6	5	1	[illegible]
Opioid Signaling Pathway	0.0204174	9%		-0.816	29	15	14	[illegible]
IL-1 Signaling	0.020893	9%		0.816	13	7	6	[illegible]
RUNX1 regulates megakaryocyte differentiation and platelet function	0.0229087	10%		0.816	9	7	2	[illegible]
IL-15 Production	0.001349	1%		0.728	19	12	7	[illegible]
Ferroptosis Signaling Pathway	0.0063096	4%		-0.728	18	10	8	[illegible]
G alpha (s) signalling events	0.0331131	13%		0.728	17	10	7	[illegible]
Surfactant metabolism	0.0001349	0%		-0.707	8	3	5	[illegible]
Toll-like Receptor Signaling	0.0004169	1%		0.707	15	12	3	[illegible]
RHO GTPases activate PKNs	0.0006166	1%		-0.707	8	3	5	[illegible]
Toll-like Receptor Cascades	0.0012882	1%		0.707	8	5	3	[illegible]
NR1H2 and NR1H3-mediated signaling	0.0263027	11%		-0.707	8	3	5	[illegible]
Glioma Signaling	0.0363078	14%		0.707	14	9	5	[illegible]
Intrinsic Pathway for Apoptosis	0.042658	16%		0.707	8	5	3	[illegible]
Gαq Signaling	0.0002291	0%		-0.688	25	13	12	[illegible]
Gap Junction Signaling	0.0072444	5%		-0.686	35	19	16	[illegible]
IL-12 Signaling and Production in Macrophages	1.549E-07	0%		0.632	39	33	6	[illegible]
TNFR1 Signaling	5.012E-05	0%		0.632	12	11	1	[illegible]
Role of CHK Proteins in Cell Cycle Checkpoint Control	0.0007762	1%		-0.632	12	11	1	[illegible]
Cell Cycle: G2/M DNA Damage Checkpoint Regulation	0.0008913	1%		0.632	11	9	2	[illegible]
Role of JAK2 in Hormone-like Cytokine Signaling	0.0128825	7%		-0.632	10	7	3	[illegible]
Neuregulin Signaling	0.0218776	10%		0.632	15	8	7	[illegible]
Role of JAK1 and JAK3 in γc Cytokine Signaling	0.0263027	11%		0.632	10	8	2	[illegible]
Cyclins and Cell Cycle Regulation	0.0457088	17%		0.632	11	7	4	[illegible]
WNK Renal Signaling Pathway	0.0093325	5%		0.577	14	9	5	[illegible]
Serotonin Receptor Signaling	0.0097724	6%		-0.577	47	24	23	[illegible]
Salvage Pathways of Pyrimidine Ribonucleotides	0.0223872	10%		0.577	13	8	5	[illegible]
P2Y Purinergic Receptor Signaling Pathway	0.030903	12%		0.577	16	9	7	[illegible]
Amyotrophic Lateral Sclerosis Signaling	0.0016596	2%		-0.5	17	10	7	[illegible]
MSP-RON Signaling in Macrophages Pathway	0.0022387	2%		-0.5	18	17	1	[illegible]
Apoptosis Signaling	1.479E-05	0%		-0.471	20	16	4	[illegible]
HEY1 Signaling Pathway	0.0436516	16%		0.471	18	11	7	[illegible]
[illegible]	[illegible]	[illegible]	[illegible]	[illegible]	[illegible]	[illegible]	[illegible]	[illegible]

9),MMP25(16.8),MMP8(3.33),MYD88(3.07),NAPSA(-4.45),NPY1R(4.22),P2RY10(4.88),PGF(6.08),PIK3CD(5.43),PIK3R5(5.29),PLA2G1B(-8.67),PLA2G5(4.63),PLCD3(7.91),PLCL2(4.46),PRKAR2B(-6.23),PRKCQ(5.2),PRKD1(-4.03),PRKD3(-4.09),PTGER4(5.32),PTH1R(4.03),RAC3(-4.23),RTKN(-3.89),S1
