# Supplementary material for: Synthetic DNA co-immunization with vaccine-aligned common consensus nucleoprotein and hemagglutinin protects mice against lethal influenza infection with a single immunization
Source: Front Immunol. 2025 Nov 26;16:1632121. doi: 10.3389/fimmu.2025.1632121 (PMC12689538; doi:10.3389/fimmu.2025.1632121)
Supplement: Supplementary Table 5 — Ingenuity Pathway analysis of pHAH1 versus naïve mice. [file DataSheet5.pdf]

Pathways	P	FDR	State	Z	N	Pos	Neg	Molecules
[illegible]	1.259E-25	0%	Inhibited	-5.842	61	7	54	[illegible]
[illegible]	7.943E-24	0%	Inhibited	-5.657	32	0	32	[illegible]
[illegible]	1E-21	0%	Inhibited	-5.196	27	0	27	[illegible]
[illegible]	2.512E-20	0%	Inhibited	-4.914	28	1	27	[illegible]
[illegible]	5.495E-06	0%	Inhibited	-4.583	21	0	21	[illegible]
[illegible]	1.096E-10	0%	Inhibited	-4.557	59	12	47	[illegible]
[illegible]	8.511E-10	0%	Inhibited	-4.481	61	15	46	[illegible]
[illegible]	5.495E-10	0%	Inhibited	-4.472	20	0	20	[illegible]
[illegible]	7.943E-11	0%	Inhibited	-4.243	17	0	17	[illegible]
[illegible]	1.072E-06	0%	Inhibited	-4.218	38	6	32	[illegible]
[illegible]	0.0006918	1%	Activated	4.111	40	10	30	[illegible]
[illegible]	1.259E-12	0%	Inhibited	-4.007	71	18	53	[illegible]
[illegible]	0.0006761	1%	Inhibited	-3.91	55	10	45	[illegible]
[illegible]	4.266E-08	0%	Inhibited	-3.889	32	5	27	[illegible]
[illegible]	0.0006761	1%	Inhibited	-3.772	31	5	26	[illegible]
[illegible]	3.311E-06	0%	Inhibited	-3.742	14	0	14	[illegible]
[illegible]	0.0001122	0%	Inhibited	-3.683	39	8	31	[illegible]
[illegible]	1.413E-07	0%	Inhibited	-3.651	30	7	23	[illegible]
[illegible]	1.479E-05	0%	Inhibited	-3.592	41	9	32	[illegible]
[illegible]	0.0001514	0%	Inhibited	-3.507	43	11	32	[illegible]
[illegible]	2.138E-08	0%	Inhibited	-3.464	12	0	12	[illegible]
[illegible]	5.129E-05	0%	Inhibited	-3.464	12	0	12	[illegible]
[illegible]	0.0008318	1%	Inhibited	-3.411	22	8	14	[illegible]
[illegible]	0.0008318	1%	Inhibited	-3.4	25	3	22	[illegible]
[illegible]	5.754E-05	0%	Inhibited	-3.317	12	0	12	[illegible]
[illegible]	2.512E-12	0%	Inhibited	-3.266	24	2	22	[illegible]
[illegible]	0.000912	1%	Inhibited	-3.207	16	2	14	[illegible]
[illegible]	6.026E-06	0%	Inhibited	-3.153	17	2	15	[illegible]
[illegible]	0.0017783	1%	Inhibited	-3.138	32	9	23	[illegible]
[illegible]	5.012E-13	0%	Inhibited	-3.13	28	3	25	[illegible]
[illegible]	0.000537	0%	Inhibited	-3.13	20	3	17	[illegible]
[illegible]	2.512E-11	0%	Inhibited	-3.053	31	3	28	[illegible]
[illegible]	6.31E-09	0%	Inhibited	-3	25	2	23	[illegible]
[illegible]	1.738E-07	0%	Inhibited	-3	19	1	18	[illegible]
[illegible]	1.995E-05	0%	Inhibited	-3	9	0	9	[illegible]
[illegible]	0.0001778	0%	Inhibited	-3	9	0	9	[illegible]
[illegible]	0.0012589	1%	Inhibited	-3	9	0	9	[illegible]
[illegible]	0.0023442	2%	Inhibited	-3	16	2	14	[illegible]
[illegible]	0.0138038	6%	Activated	3	9	9	0	[illegible]
[illegible]	2.754E-07	0%	Inhibited	-2.982	19	2	17	[illegible]
[illegible]	0.0066069	3%	Inhibited	-2.887	12	1	11	[illegible]
[illegible]	8.128E-05	0%	Inhibited	-2.828	18	2	16	[illegible]
[illegible]	0.000138	0%	Inhibited	-2.828	8	0	8	[illegible]
[illegible]	0.0005623	1%	Inhibited	-2.828	8	0	8	[illegible]
[illegible]	1.259E-09	0%	Inhibited	-2.714	12	0	12	[illegible]
[illegible]	1.738E-07	0%	Inhibited	-2.668	17	2	15	[illegible]
[illegible]	0.0001445	0%	Inhibited	-2.646	7	0	7	[illegible]
[illegible]	0.0002951	0%	Inhibited	-2.646	7	0	7	[illegible]
[illegible]	0.0024547	2%	Inhibited	-2.646	7	0	7	[illegible]
[illegible]	3.467E-09	0%	Inhibited	-2.611	35	9	26	[illegible]
[illegible]	0.0052481	3%	Inhibited	-2.596	38	5	33	[illegible]
[illegible]	1.995E-14	0%	Inhibited	-2.502	27	2	25	[illegible]
[illegible]	0.0019055	1%	Inhibited	-2.496	13	2	11	[illegible]
[illegible]	3.802E-05	0%	Inhibited	-2.449	6	0	6	[illegible]
[illegible]	0.0030903	2%	Inhibited	-2.449	6	0	6	[illegible]
[illegible]	1.202E-10	0%	Inhibited	-2.4	23	5	18	[illegible]
[illegible]	3.311E-09	0%	Inhibited	-2.324	15	0	15	[illegible]
[illegible]	7.943E-05	0%	Inhibited	-2.309	12	2	10	[illegible]
[illegible]	3.09E-06	0%	Inhibited	-2.236	20	4	16	[illegible]
[illegible]	2.754E-05	0%	Inhibited	-2.236	6	0	6	[illegible]
[illegible]	0.0008913	1%	Inhibited	-2.236	5	0	5	[illegible]
[illegible]	0.0014125	1%	Inhibited	-2.183	17	4	13	[illegible]
[illegible]	0.0019498	1%	Inhibited	-2.183	17	6	11	[illegible]
[illegible]	0.0012589	1%	Inhibited	-2.121	9	2	7	[illegible]
[illegible]	0.0052481	3%	Inhibited	-2.121	8	1	7	[illegible]
[illegible]	0.0060256	3%	Inhibited	-2.121	12	3	9	[illegible]
[illegible]	7.762E-05	0%	Inhibited	-2.111	11	2	9	[illegible]
[illegible]	0.0004074	0%	Inhibited	-2.111	10	0	10	[illegible]
[illegible]	0.0030903	2%	Inhibited	-2.111	11	2	9	[illegible]
[illegible]	0.0037154	2%	Inhibited	-2.111	11	2	9	[illegible]
[illegible]	0.0044668	3%	Inhibited	-2.111	11	2	9	[illegible]
[illegible]	1.905E-06	0%		-2	16	2	14	[illegible]
[illegible]	4.467E-05	0%		-2	5	0	5	[illegible]
[illegible]	0.0001413	0%		-2	5	0	5	[illegible]
[illegible]	0.0001445	0%		-2	4	0	4	[illegible]
[illegible]	0.0001622	0%		-2	8	0	8	[illegible]
[illegible]	0.001	1%		-2	4	0	4	[illegible]
[illegible]	0.0027542	2%		-2	4	0	4	[illegible]
[illegible]	0.0040738	2%		-2	4	0	4	[illegible]
[illegible]	0.0107152	5%		-2	4	0	4	[illegible]
[illegible]	0.0177828	7%		-2	4	0	4	[illegible]
[illegible]	0.0177828	7%		-2	4	0	4	[illegible]
[illegible]	0.0190546	8%		-2	9	2	7	[illegible]
[illegible]	1.318E-09	0%		-1.964	22	2	20	[illegible]
[illegible]	1.514E-09	0%		-1.961	26	5	21	[illegible]
[illegible]	0.0120226	5%		-1.941	14	4	10	[illegible]
[illegible]	0.016197	8%		-1.941	13	3	10	[illegible]
[illegible]	0.0003311	0%		-1.897	10	1	9	[illegible]
[illegible]	0.0026303	2%		-1.897	12	2	10	[illegible]
[illegible]	7.762E-07	0%		-1.89	28	7	21	[illegible]
[illegible]	0.0001175	0%		-1.89	10	2	8	[illegible]
[illegible]	0.0036308	2%		-1.89	10	4	6	[illegible]
[illegible]	0.0066069	3%		-1.89	8	2	6	[illegible]
[illegible]	0.0154882	7%		1.89	9	3	6	[illegible]
[illegible]	0.0371535	13%		-1.89	9	3	6	[illegible]
[illegible]	8.318E-06	0%		-1.886	20	7	13	[illegible]
[illegible]	1.66E-08	0%		-1.8	24	3	21	[illegible]
[illegible]	3.548E-05	0%		-1.789	20	4	16	[illegible]
[illegible]	0.0138038	6%		-1.732	12	2	10	[illegible]
[illegible]	1.82E-06	0%		1.706	22	2	20	[illegible]
[illegible]	3.236E-05	0%		-1.706	22	8	14	[illegible]
[illegible]	0.0023442	2%		-1.698	19	7	12	[illegible]
[illegible]	8.128E-05	0%		-1.667	10	1	9	[illegible]
[illegible]	0.000871	1%		-1.667	9	4	5	[illegible]
[illegible]	0.0077625	4%		-1.667	10	2	8	[illegible]
[illegible]	0.0001622	0%		-1.633	8	1	7	[illegible]
[illegible]	0.0008511	1%		-1.633	7	0	7	[illegible]
[illegible]	0.0034674	2%		-1.633	6	1	5	[illegible]
[illegible]	0.0114815	5%		-1.633	6	0	6	[illegible]
[illegible]	0.0006607	1%		-1.5	16	5	11	[illegible]
[illegible]	0.0123027	5%		-1.5	16	5	11	[illegible]
[illegible]	0.0295121	11%		-1.5	16	5	11	[illegible]
[illegible]	2.512E-15	0%		-1.48	37	4	33	[illegible]
[illegible]	6.166E-05	0%		-1.414	18	3	15	[illegible]
Kinesins	0.001	1%		-1.414	8	2	6	[illegible]
[illegible]	0.0027542	2%		-1.414	9	3	6	[illegible]
[illegible]	0.0081283	4%		-1.414	10	3	7	[illegible]
[illegible]	0.0104713	5%		1.414	9	3	6	[illegible]
[illegible]	5.37E-05	0%		-1.342	19	3	16	[illegible]
[illegible]	9.333E-05	0%		-1.342	20	7	13	[illegible]
[illegible]	0.0010965	1%		-1.342	5	1	4	[illegible]
[illegible]	0.0019055	1%		-1.342	5	1	4	[illegible]
[illegible]	0.0036308	2%		-1.342	5	1	4	[illegible]
[illegible]	0.01	5%		-1.342	10	4	6	[illegible]
[illegible]	0.0380189	14%		-1.342	5	1	4	[illegible]
[illegible]	0.0489779	16%		-1.342	5	1	4	[illegible]
[illegible]	0.0489779	16%		-1.291	16	4	12	[illegible]
[illegible]	0.0006761	1%		-1.265	12	4	8	[illegible]
[illegible]	0.0012882	1%		-1.265	12	3	9	[illegible]
[illegible]	0.0338844	13%		-1.213	17	7	10	[illegible]
[illegible]	6.761E-05	0%		1.155	13	1	12	[illegible]
[illegible]	0.0005754	1%		-1.155	15	7	8	[illegible]
[illegible]	0.0048978	3%		-1.155	12	5	7	[illegible]
[illegible]	7.586E-06	0%		1.134	7	5	2	[illegible]
[illegible]	0.0012589	1%		-1.134	8	3	5	[illegible]
[illegible]	0.0048978	3%		1.134	7	2	5	[illegible]
[illegible]	0.006166	3%		-1.134	8	3	5	[illegible]
[illegible]	0.0069183	3%		-1.134	7	2	5	[illegible]
[illegible]	0.0081283	4%		-1.134	8	3	5	[illegible]
[illegible]	3.802E-09	0%		-1.121	39	12	27	[illegible]
[illegible]	6.026E-08	0%		-1.091	21	2	19	[illegible]
[illegible]	0.0002951	0%		-1.069	14	5	9	[illegible]
[illegible]	0.0004786	0%		-1	11	3	8	[illegible]
[illegible]	0.0012589	1%		-1	9	1	8	[illegible]
[illegible]	0.0016982	1%		-1	4	1	3	[illegible]
[illegible]	0.0030903	2%		1	11	4	7	[illegible]
[illegible]	0.0141254	6%		-1	4	1	3	[illegible]
[illegible]	0.0158489	7%		-1	6	3	3	[illegible]
[illegible]	0.0165959	7%		-1	7	2	5	[illegible]
[illegible]	0.017378	7%		-1	16	6	10	[illegible]
[illegible]	0.0288403	11%		1	5	1	4	[illegible]
[illegible]	0.0288403	11%		-1	8	3	5	[illegible]
[illegible]	0.0301995	11%		-1	11	3	8	[illegible]
[illegible]	0.0354813	13%		-1	4	0	4	[illegible]
[illegible]	0.0436516	15%		-1	5	2	3	[illegible]
[illegible]	0.0457088	16%		-1	8	3	5	[illegible]
[illegible]	0.0069183	3%		0.943	18	8	10	[illegible]
[illegible]	0.0269153	11%		-0.905	13	5	8	[illegible]
[illegible]	0.0018621	1%		0.853	22	13	9	[illegible]
[illegible]	0.000123	0%		-0.832	14	4	10	[illegible]
[illegible]	0.0070795	4%		0.832	13	5	8	[illegible]
[illegible]	2.692E-05	0%		-0.816	8	1	7	[illegible]
[illegible]	0.0032359	2%		-0.816	8	4	4	[illegible]
[illegible]	0.0043652	2%		-0.816	6	1	5	[illegible]
[illegible]	0.0069183	3%		-0.816	7	1	6	[illegible]
[illegible]	0.0072444	4%		-0.816	5	1	4	[illegible]
[illegible]	0.0144544	6%		0.816	7	5	2	[illegible]
[illegible]	0.0223872	9%		-0.816	5	0	5	[illegible]
[illegible]	0.0288403	11%		-0.816	9	4	5	[illegible]
[illegible]	0.0346737	13%		0.816	7	4	3	[illegible]
[illegible]	0.0389045	14%		-0.816	7	3	4	[illegible]
[illegible]	0.0489779	16%		-0.816	6	1	5	[illegible]
[illegible]	4.074E-05	0%		-0.784	31	11	20	[illegible]
[illegible]	0.0005129	0%		-0.728	17	6	11	[illegible]
[illegible]	0.0002291	0%		-0.707	9	3	6	[illegible]
[illegible]	0.0013804	1%		-0.707	9	3	6	[illegible]
[illegible]	0.0052481	3%		-0.707	8	1	7	[illegible]
[illegible]	0.0093325	4%		0.707	9	4	5	[illegible]
[illegible]	2.512E-05	0%		0.632	11	3	8	[illegible]
[illegible]	0.0199526	8%		-0.632	10	2	8	[illegible]
[illegible]	2.57E-05	0%		0.626	23	9	14	[illegible]
[illegible]	3.162E-06	0%		-0.577	12	3	9	[illegible]
[illegible]	0.0097724	5%		-0.577	12	3	9	[illegible]
[illegible]	0.0102329	5%		-0.577	12	5	7	[illegible]
[illegible]	0.0389045	14%		0.577	12	3	9	[illegible]
[illegible]	0.006166	3%		0.447	5	2	3	[illegible]
[illegible]	0.0077625	4%		-0.447	4	0	4	[illegible]
[illegible]	0.0162181	7%		0.447	5	3	2	[illegible]
[illegible]	0.0162181	7%		0.447	5	3	2	[illegible]
[illegible]	0.0162181	7%		-0.447	8	2	6	[illegible]
[illegible]	0.0245471	10%		-0.447	5	2	3	[illegible]
[illegible]	0.0295121	11%		0.447	6	2	4	[illegible]
[illegible]	0.030903	11%		-0.447	5	2	3	[illegible]
[illegible]	0.040738	14%		-0.447	5	2	3	[illegible]
[illegible]	0.0467735	16%		-0.447	6	2	4	[illegible]
[illegible]	0.0363078	13%		0.426	22	8	14	[illegible]
[illegible]	0.0074131	4%		-0.378	7	2	5	[illegible]
[illegible]	0.0199526	8%		-0.378	9	5	4	[illegible]
[illegible]	0.020893	9%		0.378	9	4	5	[illegible]
[illegible]	0.0056234	3%		-0.333	13	6	7	[illegible]
[illegible]	0.0060256	3%		-0.333	9	2	7	[illegible]
[illegible]	0.0263027	10%		0.333	9	5	4	[illegible]
[illegible]	0.0436516	15%		-0.333	9	3	6	[illegible]
[illegible]	1.995E-05	0%		0.302	12	3	9	[illegible]
[illegible]	8.71E-05	0%		-0.302	12	4	8	[illegible]
[illegible]	0.000195	0%		-0.302	17	4	13	[illegible]
[illegible]	0.0093325	4%		0.302	16	9	7	[illegible]
[illegible]	0.0213796	9%		-0.302	11	4	7	[illegible]
[illegible]	1.23E-05	0%		0.258	15	3	12	[illegible]
[illegible]	1.549E-07	0%		0	19	2	17	[illegible]
[illegible]	1.738E-05	0%		0	8	2	6	[illegible]
[illegible]	0.0010233	1%		0	10	2	8	[illegible]
[illegible]	0.0014125	1%		0	16	10	6	[illegible]
[illegible]	0.0028184	2%		0	16	7	9	[illegible]
[illegible]	0.0041687	2%		0	4	1	3	[illegible]
[illegible]	0.0052481	3%		0	8	4	4	[illegible]
[illegible]	0.0144544	6%		0	10	4	6	[illegible]
[illegible]	0.020893	9%		0	6	3	3	[illegible]
[illegible]	0.0239883	9%		0	7	4	3	[illegible]
[illegible]	0.0263027	10%		0	5	2	3	[illegible]
[illegible]	0.0316228	12%		0	10	4	6	[illegible]
[illegible]	0.0331131	12%		0	10	4	6	[illegible]
[illegible]	0.0416869	14%		0	12	7	5	[illegible]
[illegible]	0.0457088	16%		0	14	4	10	[illegible]
[illegible]	0.0489779	16%		0	7	3	4	[illegible]

(3.07),SHH(3.22),SMAD9(6.04)
