# Supplementary figures and images for: Synthetic DNA co-immunization with vaccine-aligned common consensus nucleoprotein and hemagglutinin protects mice against lethal influenza infection with a single immunization
Source: Front Immunol. 2025 Nov 26;16:1632121. doi: 10.3389/fimmu.2025.1632121 (PMC12689538; doi:10.3389/fimmu.2025.1632121)

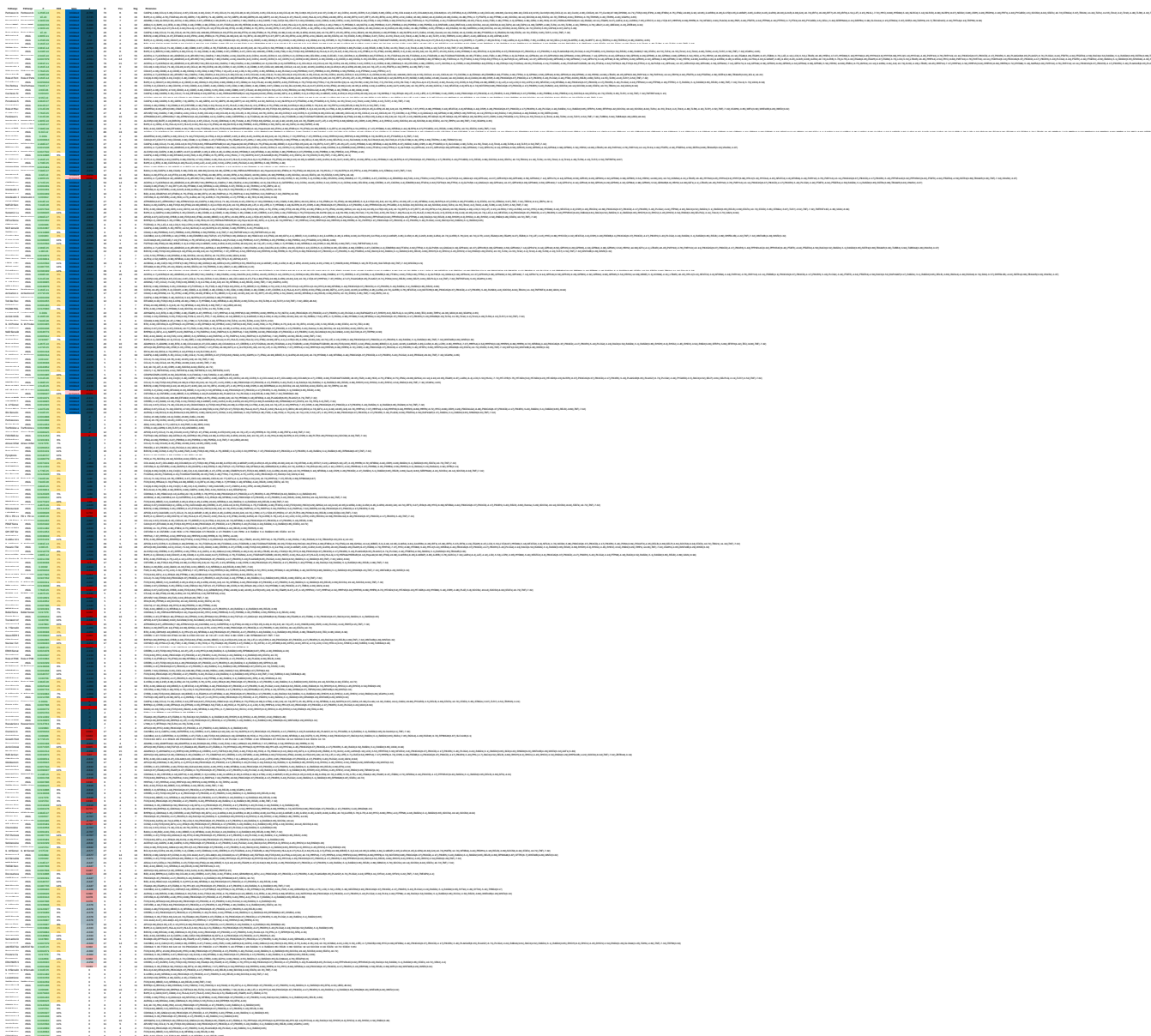

Supplement: Supplementary Table 6 — Ingenuity Pathway analysis of pVACC-NPH3 versus naïve mice. [file DataSheet6.pdf]

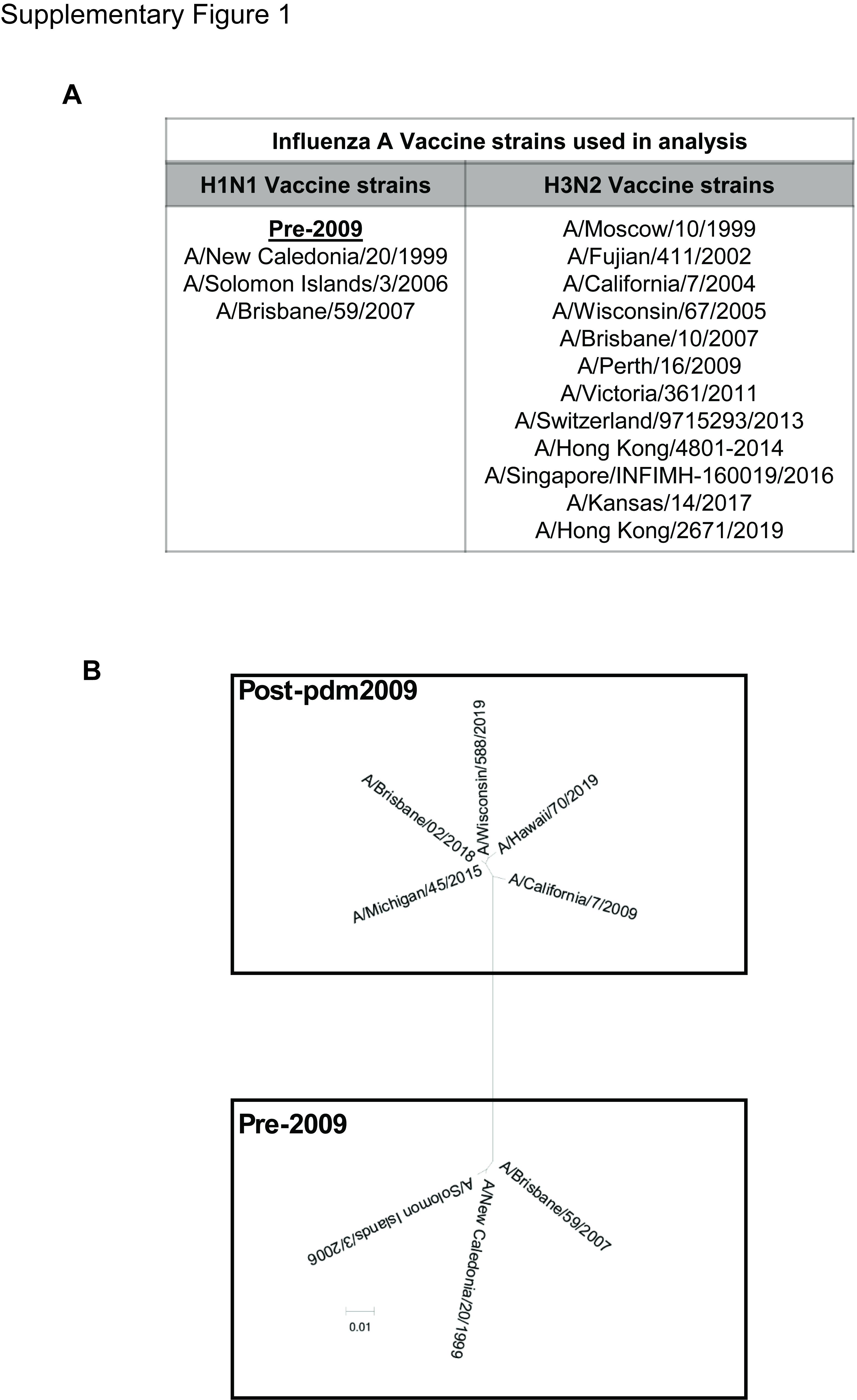

Supplement: Supplementary Figure 1 — (A)The H1N1 and H3N2 vaccine strains included in the generation of the vaccine pVACC-NPH1 and pVACC-NPH3. (B) A phylogenetic tree [file Image1.tif]

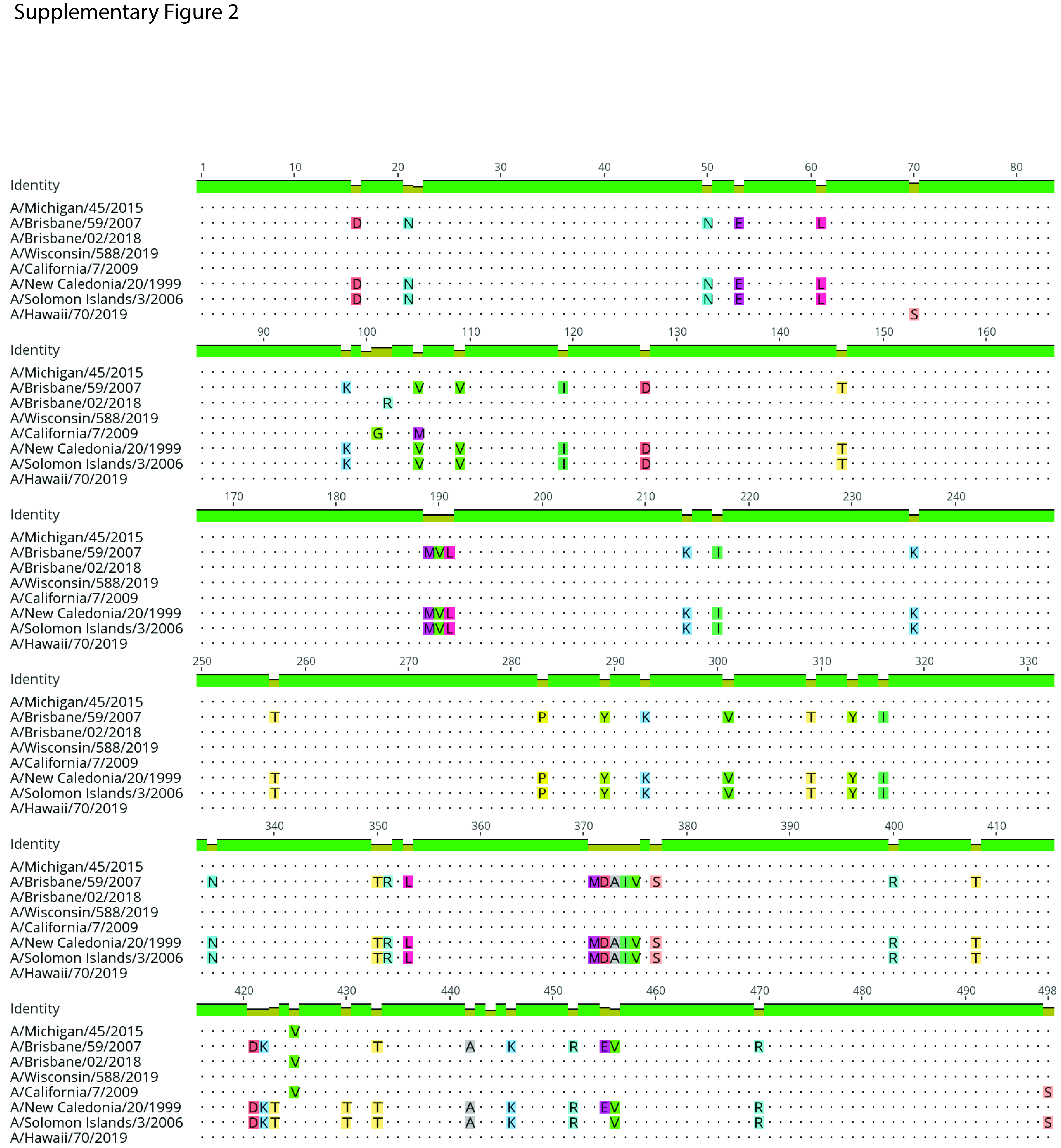

Supplement: Supplementary Figure 2 — Multiple sequence alignment of NP proteins of seasonal A/H1N1 vaccine strains, performed in Geneious Prime. Dots represent complete identity of the residue in all the sequences. The green bar on top represents the level of conservation of the residues between strains, deep green indicates most conserved, yellow indicates a poorly conserved position. [file Image2.tif]

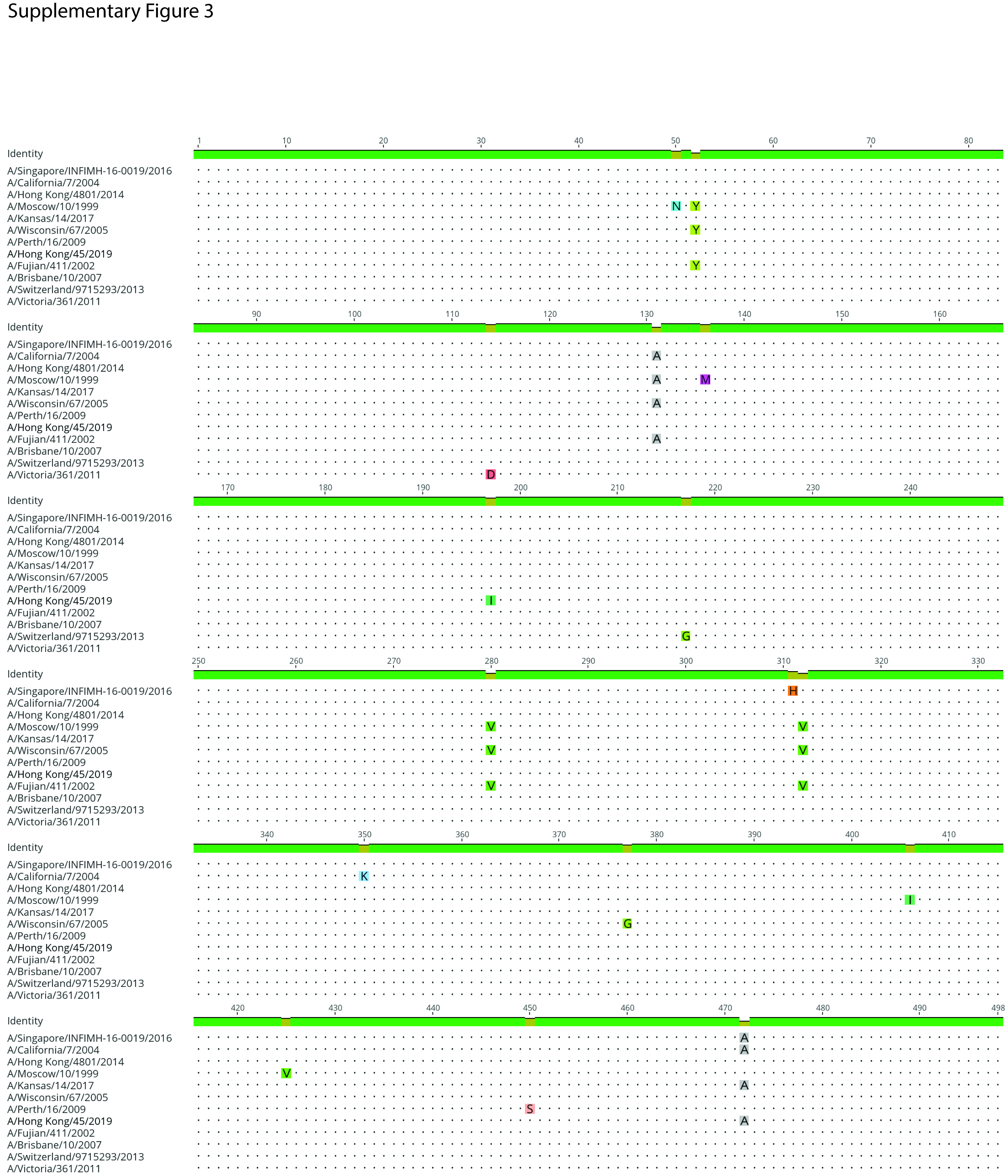

Supplement: Supplementary Figure 3 — Multiple sequence alignment of NP proteins of seasonal A/H3N2 vaccine strains, performed in Geneious Prime. Dots represent complete identity of the residue in all the sequences. The green bar on top represents the level of conservation of the residues between strains, deep green indicates most conserved, yellow indicates a poorly conserved position. [file Image3.tif]

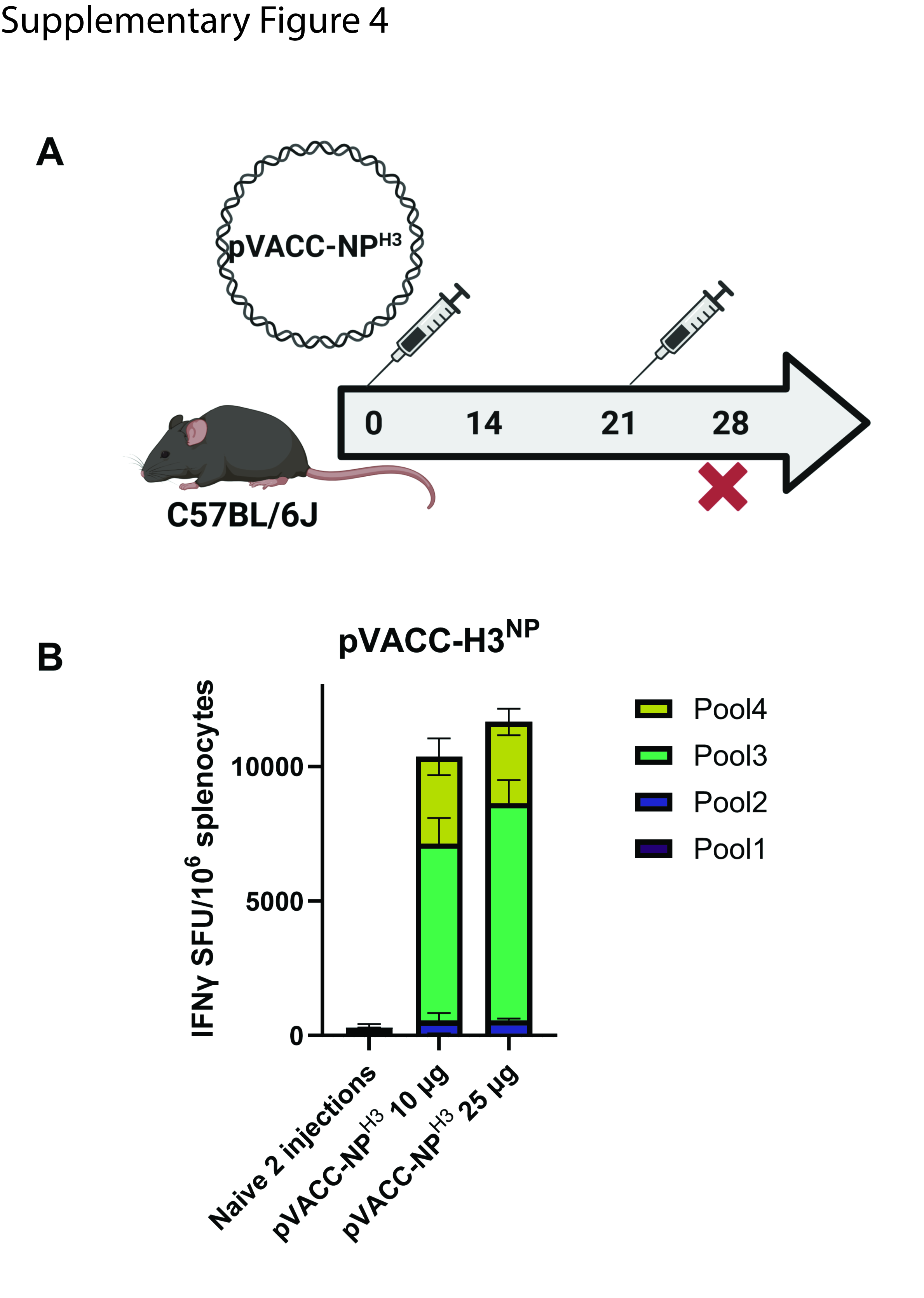

Supplement: Supplementary Figure 4 — A 2-injection regimen of VACC-NPH3 induces robust immune responses in C57BL/6J mice. C57BL/6J mice were immunized with 10µg or 25µg of pVACC-NPH3 plasmid at a 21-day interval and euthanized seven days post-immunization for cellular analyses (A). IFNγ spot-forming units (SFUs) in spleens following stimulation with H3NP peptides (n=5 mice per group) (B). [file Image4.tif]

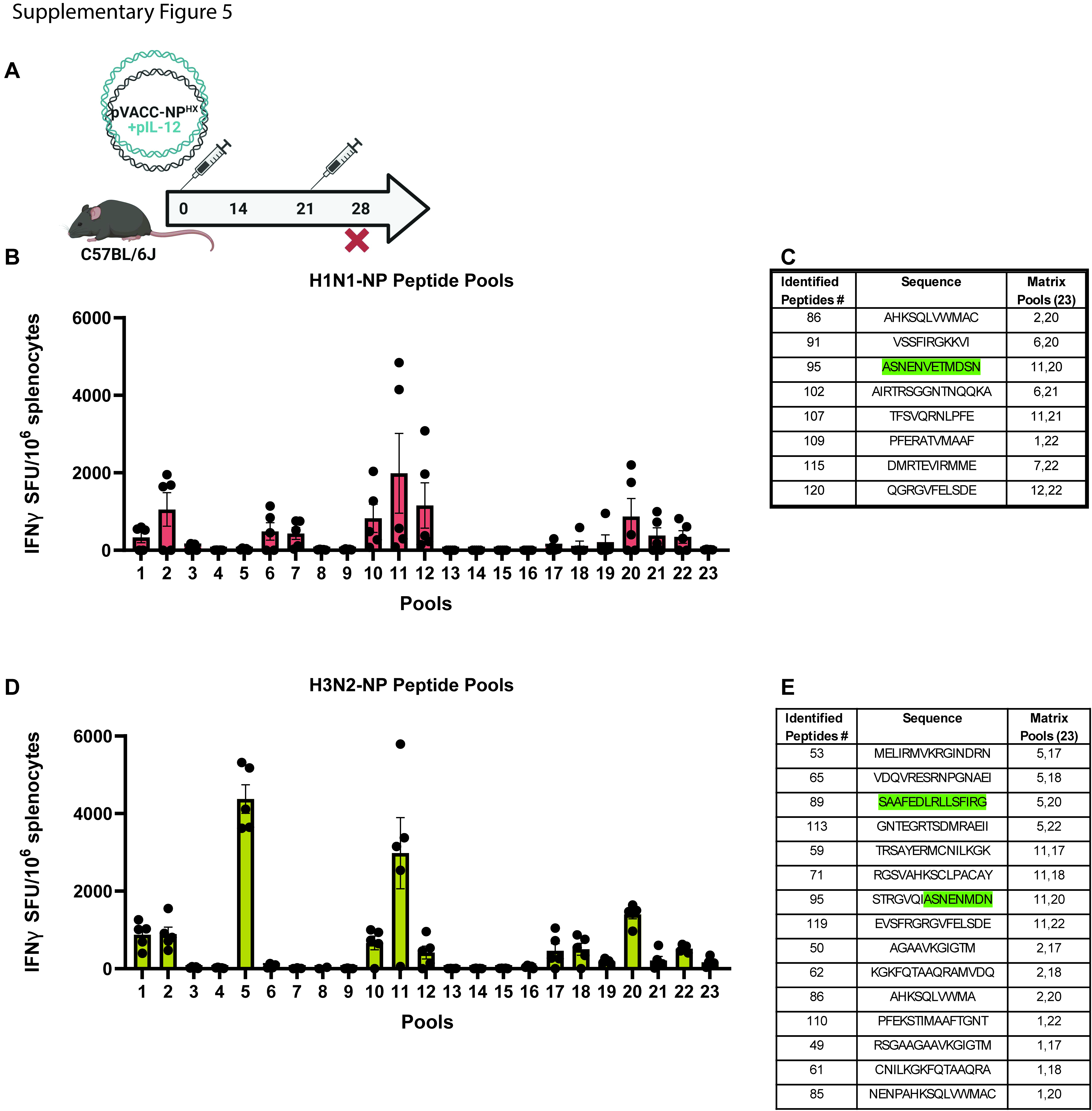

Supplement: Supplementary Figure 5 — Identification of pVACC-NPX immunodominant T cell epitopes. (A) C57BL/6J mice were immunized twice, separated by three weeks with 10μg of pVACC-NPX. Matrix peptide pools were used to stimulate isolated splenocytes. (B) H1N1-NP specific IFNγ secretion as measured by ELISpot. (C) Identified H1N1-NP immunodominant peptides. (D) H3N2-NP specific IFNγ secretion as measured by ELISpot. (E) Identified H3N2-NP immunodominant peptides. Data are representative of one experiment with n=5/group. Symbols represent the average of duplicate assays per animal, bars represent group mean, error bars represent SEM. [file Image5.tif]

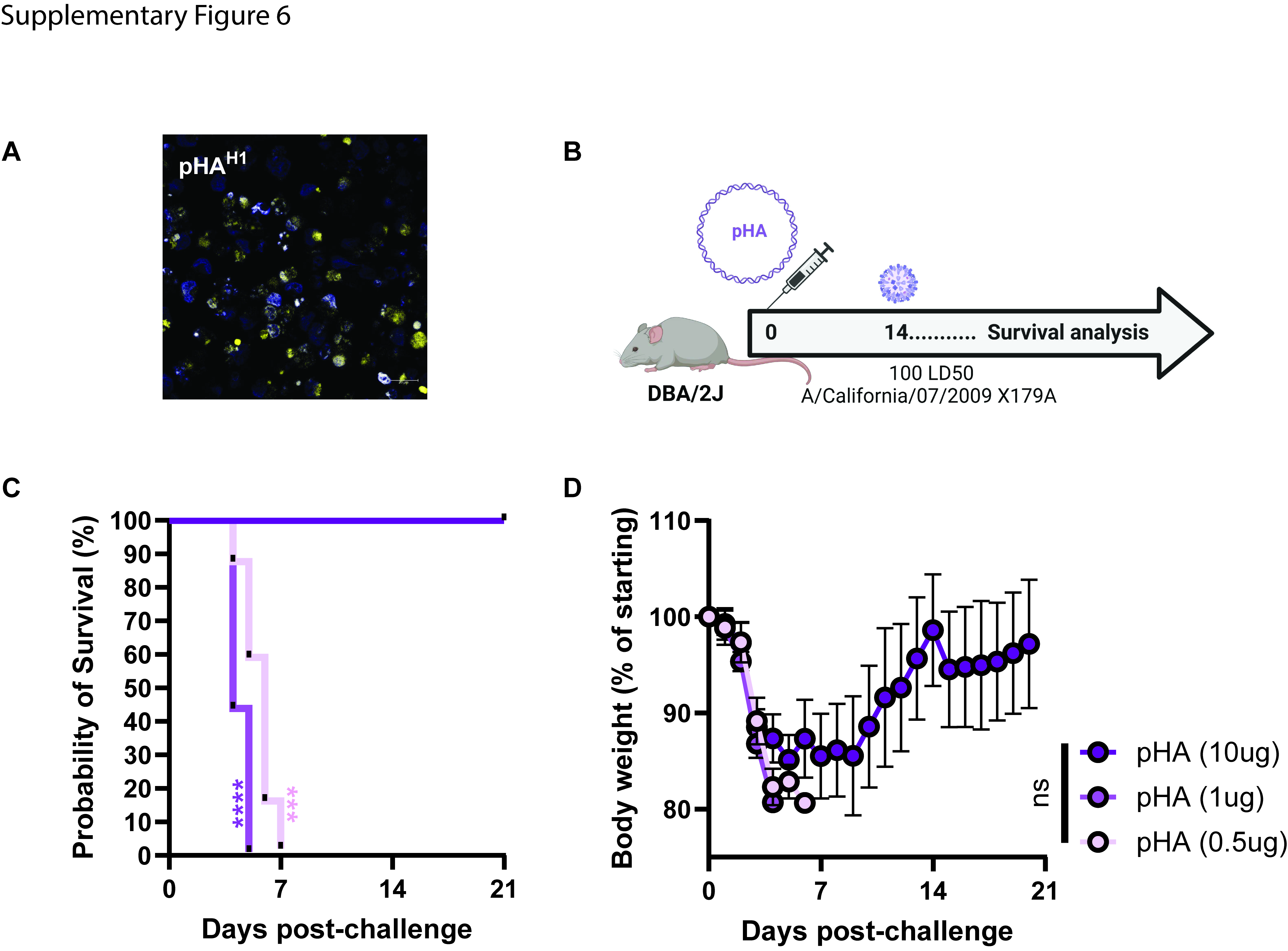

Supplement: Supplementary Figure 6 — pHAH1 is sub-protective in high-dose IAV challenge: (A) Immunofluorescence staining of HEK293T cells transfected with pHAH1 plasmid and stained for IAV-NP. (B) Mice were immunized once with 10μg, 1μg, or 0.5μg of plasmid-encoded A/California/07/2009 HA (pHAH1) and challenged with 100 LD50 of Ca09-X179A virus fourteen days later. (C) Survival probability. (D) Body weights as percent of starting weight. Data are representative of one experiment with n=10/group. Symbols represent group mean, error bars represent SD. ***p<0.001, ****p<0.0001 Mantel-Cox Log-rank test (C) ns = not significant by Dunnett’s multiple comparison test (D). [file Image6.tif]

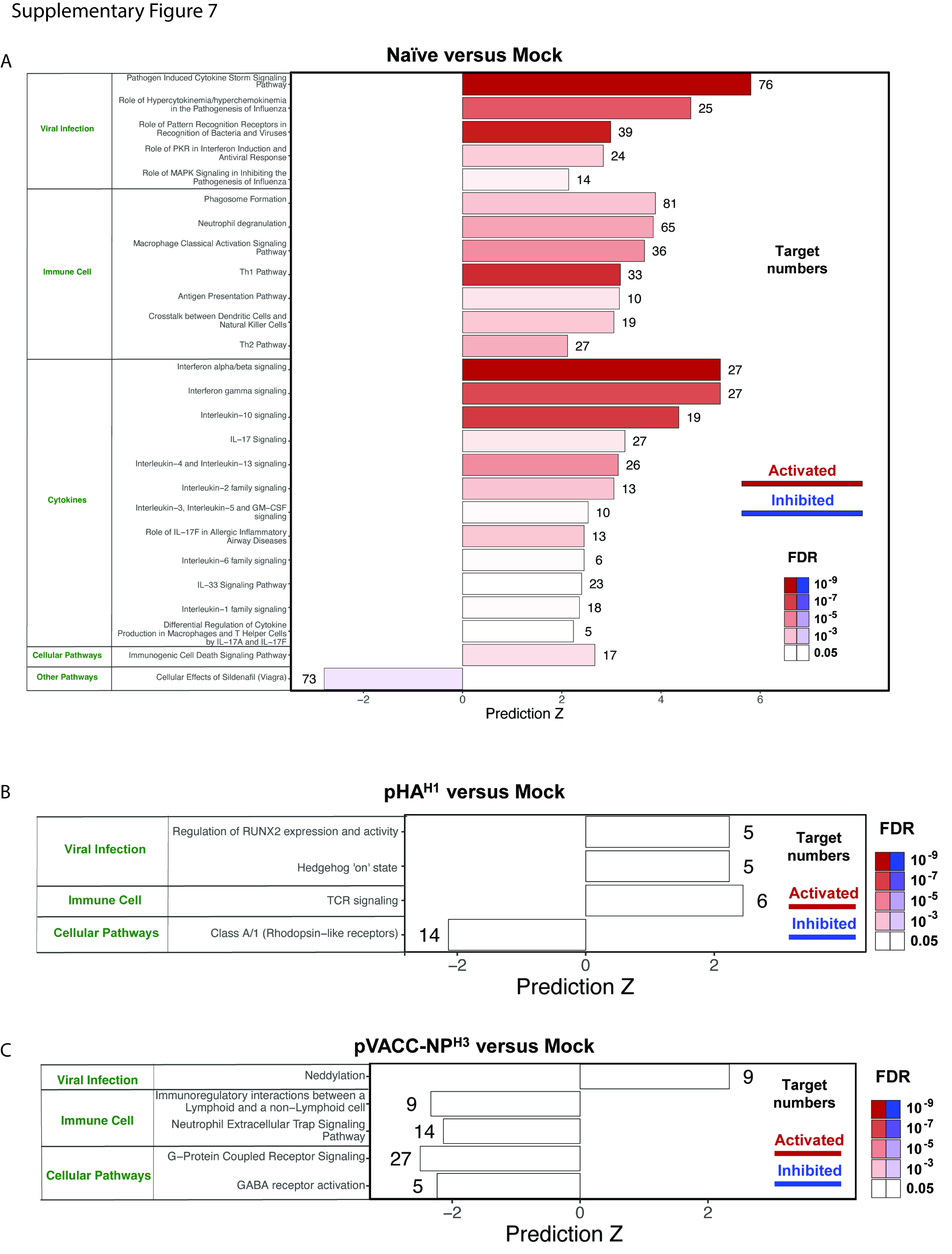

Supplement: Supplementary Figure 7 — VACC-NPX immunogens induce robust and durable cellular responses in vivo. (A) Female C57BL/6J mice were immunized twice separated by three weeks and mice were rested for 200 days (six months) with 10 μg of pVACC-NPH1 or pVACC-NPH3 alone, or co-immunized with pVACC-NPx and 0.5μg of plasmid-encoded IL-12 (+pIL-12). (B) NPH1-specific IFNγ spot-forming units (SFU) in spleens and (D) lungs. (C) NPH3-specific IFNγ spot-forming units (SFU) in spleens (E) and lungs. Data are representative of one experiment with n=5/group. Bars represent the mean; error bars represent SEM (C-J) or SD (K-R). **p<0.01, ***p<0.001, ****p<0.0001 by Two-way ANOVA. [file Image7.tif]

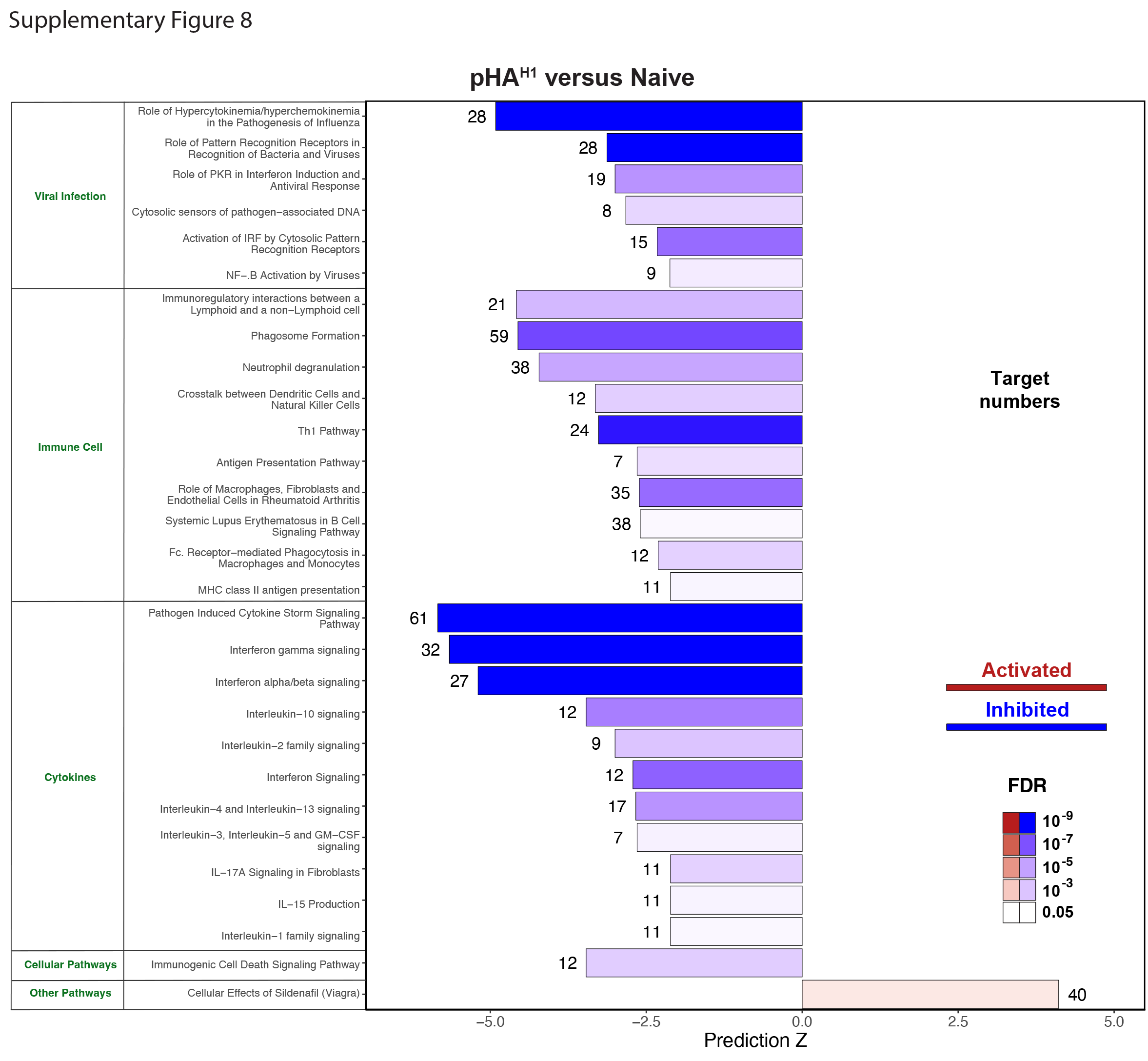

Supplement: Supplementary Figure 8 — Ingenuity pathway analysis (IPA) of versus Mock (unvaccinated, uninfected). IPA was performed on the total gene expression dataset and further categorized into subsets involved with viral infection, immune cells, cytokines, cellular pathways, and other pathways. Bar charts comparing the number of activated and inhibited pathways are shown for (A) naïve versus mock, (B) pHAH1 versus mock, and (C) pVACC-NPH3 versus mock groups. Groups were first filtered by Z-score >2.0 and a false discovery rate of 0.05. All groups have significant p-values <0.05. [file Image8.tif]

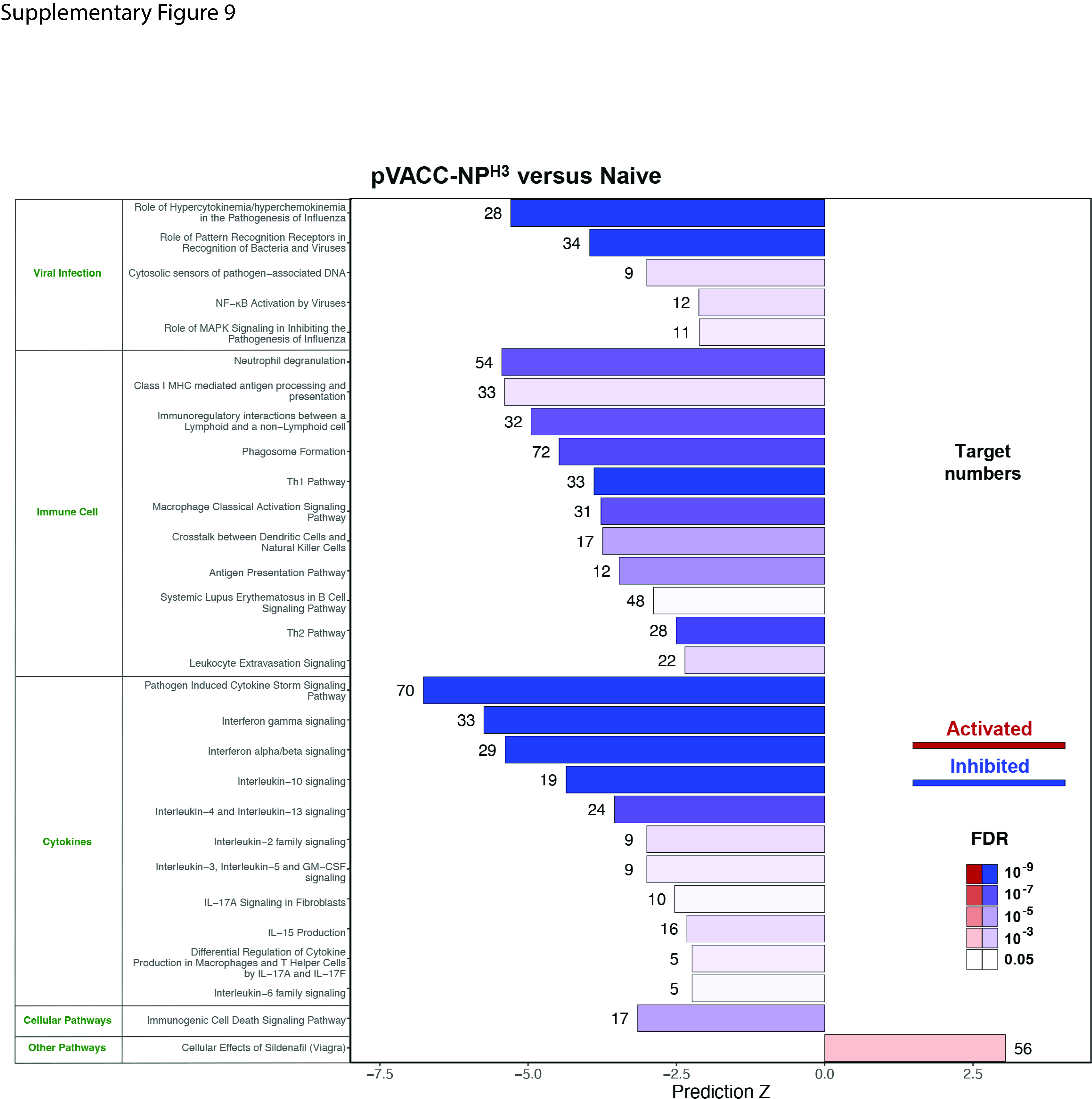

Supplement: Supplementary Figure 9 — Ingenuity pathway analysis (IPA) of pHAH1 versus Naïve. IPA was performed on the total gene expression dataset and further categorized into subsets involved with viral infection, immune cells, cytokines, cellular pathways, and other pathways. Bar charts comparing the number of activated and inhibited pathways are shown for pHAH1 versus naïve. Groups were first filtered by Z-score >2.0 and a false discovery rate of 0.05. [file Image9.tif]

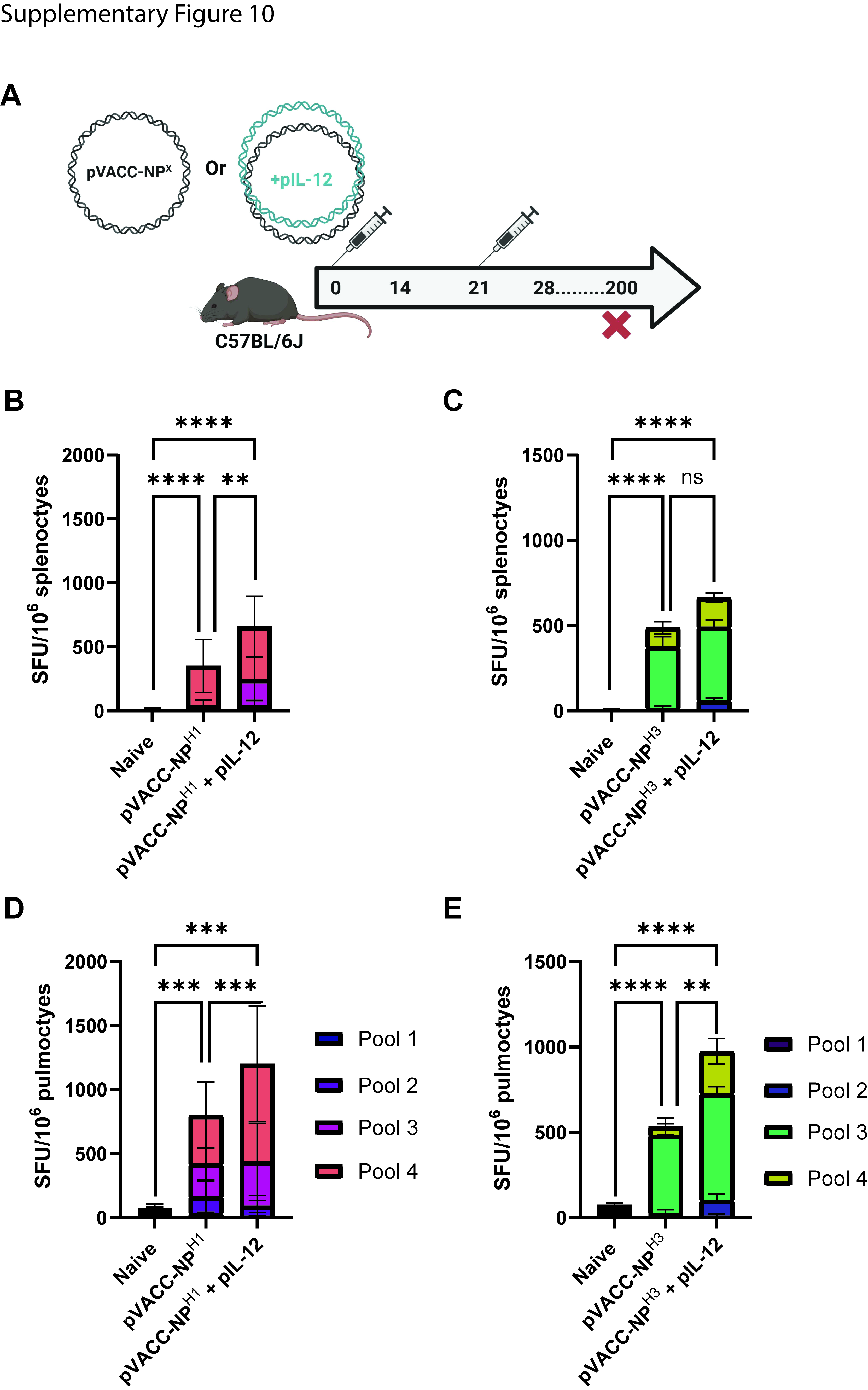

Supplement: Supplementary Figure 10 — Ingenuity pathway analysis (IPA) of pVACC-NPH3 versus Naïve. IPA was performed on the total gene expression dataset and further categorized into subsets involved with viral infection, immune cells, cytokines, cellular pathways, and other pathways. Bar charts comparing the number of activated and inhibited pathways are shown for pVACC-NPH3 versus naïve. Groups were first filtered by Z-score >2.0 and a false discovery rate of 0.05. [file Image10.tif]

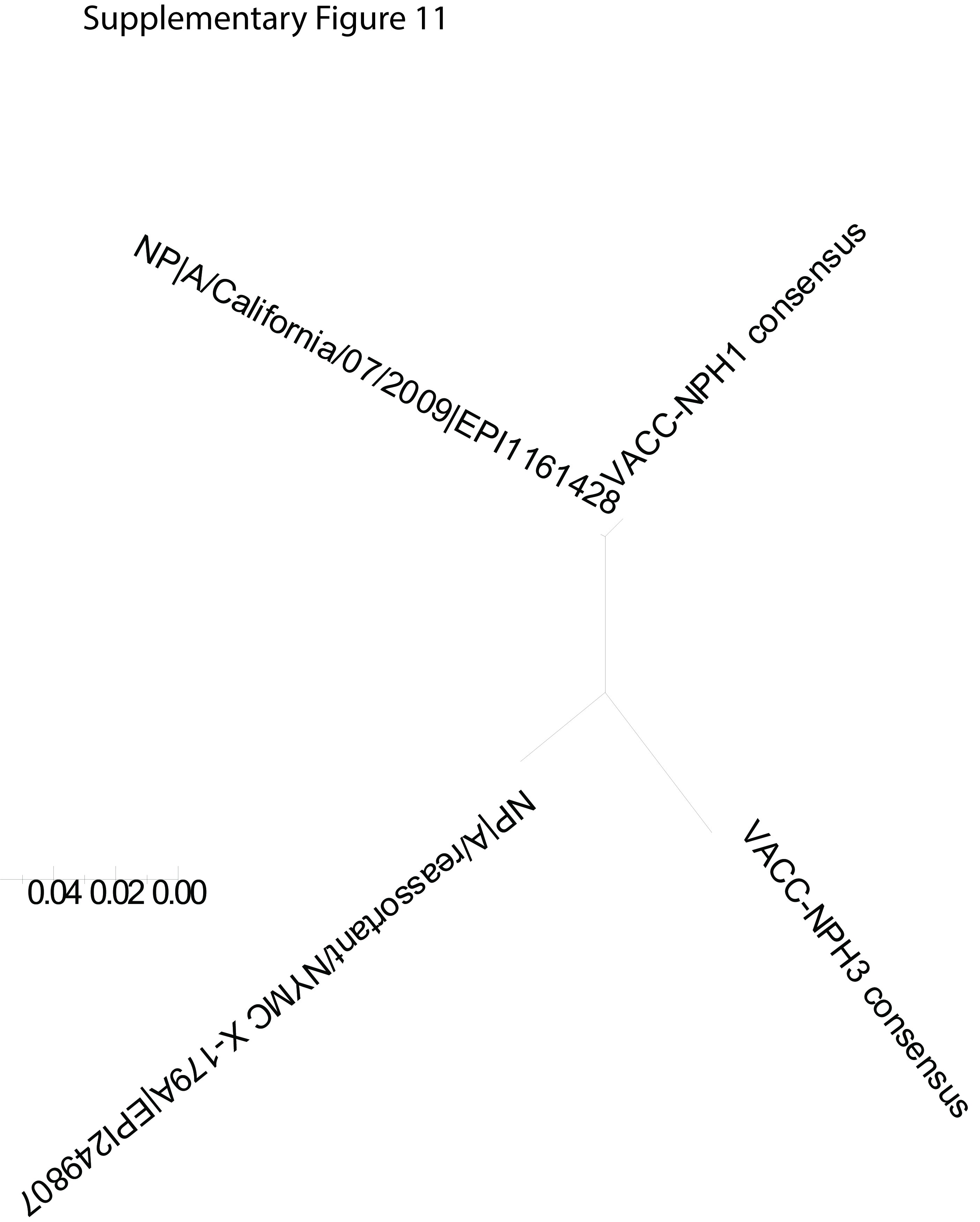

Supplement: Supplementary Figure 11 — Phylogenetic analysis comparing pVACC-NPX immunogens with A/California/07/2009 and Ca09-X179A (PR8) NP proteins. [file Image11.tif]
