# Supplementary material for: Synthetic DNA co-immunization with vaccine-aligned common consensus nucleoprotein and hemagglutinin protects mice against lethal influenza infection with a single immunization
Source: Front Immunol. 2025 Nov 26;16:1632121. doi: 10.3389/fimmu.2025.1632121 (PMC12689538; doi:10.3389/fimmu.2025.1632121)
Supplement: Supplementary Table 1 — Influenza NP peptides and pools for ICS and ELISpot assays. (A) pVACC-NPH1 peptides and pools. (B) pVACC-NPH3 peptides and pools. (C) Peptide matrix pool design schematic. [file DataSheet1.pdf]

Supplementary Table 1

A

pVACC-NP^{H1} Peptides

Peptide ID	Peptide Sequence	Pool #
1	SQGTKRSYEQMETGG	1
2	KRSYEQMETGGERQD	1
3	EQMETGGERQDTTEI	1
4	TGGERQDTTEIRASV	1
5	RQDTTEIRASVGRMI	1
6	TEIRASVGRMIGGIG	1
7	ASVGRMIGGIGRFYI	1
8	RMIGGIGRFYIQMCT	1
9	GIGRFYIQMCTELKL	1
10	FYIQMCTELKLSDYD	1
11	MCTELKLSDYDGRLI	1
12	LKLSDYDGRLIQNSI	1
13	DYDGRLIQNSITIER	1
14	RLIQNSITIERMVLS	1
15	NSITIERMVLSAFDE	1
16	IERMVLSAFDERRNK	1
17	VLSAFDERRNKYLEE	1
18	FDERRNKYLEEHPSA	1
19	RNKYLEEHPSAGKDP	1
20	LEEHPSAGKDPKKTG	1
21	PSAGKDPKKTGGPIY	1
22	KDPKKTGGPIYRRID	1
23	KTGGPIYRRIDGKWT	1
24	PIYRRIDGKWTRELI	1
25	RIDGKWTRELILYDK	1

Peptide ID	Peptide Sequence	Pool #
26	KWTRELILYDKEEIR	2
27	ELILYDKEEIRRVWR	2
28	YDKEEIRRVWRQANN	2
29	EIRRVWRQANNGEDA	2
30	VWRQANNGEDATAGL	2
31	ANNGEDATAGLTHIM	2
32	EDATAGLTHIMIWHS	2
33	AGLTHIMIWHSNLND	2
34	HIMIWHSNLNDATYQ	2
35	WHSNLNDATYQRTRA	2
36	LNDATYQRTRALVRT	2
37	TYQRTRALVRTGMDP	2
38	TRALVRTGMDPRMCS	2
39	VRTGMDPRMCSLMQG	2
40	MDPRMCSLMQGSTLP	2
41	MCSLMQGSTLPRRSG	2
42	MQGSTLPRRSGAAGA	2
43	TLPRRSGAAGAAVKG	2
44	RSGAAGAAVKGVGTI	2
45	AGAAVKGVGTIAMEL	2
46	VKGVGTIAMELIRMI	2
47	GTIAMELIRMIKRGI	2
48	MELIRMIKRGINDRN	2
49	RMIKRGINDRNFWRG	2
50	RGINDRNFWRGENGR	2
51	DRNFWRGENGRRTRV	2
52	WRGENGRRTRVAYER	2
53	NGRRTRVAYERMCNI	2
54	TRVAYERMCNILKGK	2
55	YERMCNILKGKFQTA	2

Peptide ID	Peptide Sequence	Pool #
56	CNILKGKFQTAAQRA	3
57	KGKFQTAAQRAMMDQ	3
58	QTAAQRAMMDQVRES	3
59	QRAMMDQVRESRNPG	3
60	MDQVRESRNPGNAEI	3
61	RESRNPGNAEIEDLI	3
62	NPGNAEIEDLIFLAR	3
63	AEIEDLIFLARSALI	3
64	DLIFLARSALILRGS	3
65	LARSALILRGSVAHK	3
66	ALILRGSVAHKSCLP	3
67	RGSVAHKSCLPACVY	3
68	AHKSCLPACVYGLAV	3
69	CLPACVYGLAVASGH	3
70	CVYGLAVASGHDFER	3
71	LAVASGHDFEREGYS	3
72	SGHDFEREGYSLVGI	3
73	FEREGYSLVGIDPFK	3
74	GYSLVGIDPFKLLQN	3
75	VGIDPFKLLQNSQVV	3
76	PFKLLQNSQVVSLMR	3
77	LQNSQVVSLMRPNEN	3
78	QVVSLMRPNENPAHK	3
79	LMRPNENPAHKSQLV	3
80	NENPAHKSQLVWMAC	3
81	AHKSQLVWMACHSAA	3
82	QLVWMACHSAAFEDL	3
83	MACHSAAFEDLRVSS	3
84	SAAFEDLRVSSFIRG	3
85	EDLRVSSFIRGKKVI	3
86	VSSFIRGKKVIPRGK	3
87	IRGKKVIPRGKLSTR	3
88	KVIPRGKLSTRGVQI	3
89	RGKLSTRGVQIASNE	3

Peptide ID	Peptide Sequence	Pool #
90	STRGVQIASNENVET	4
91	VQIASNENVETMDSN	4
92	SNENVETMDSNTLEL	4
93	VETMDSNTLELRSRY	4
94	DSNTLELRSRYWAIR	4
95	LELRSRYWAIRTRSG	4
96	SRYWAIRTRSGGNTN	4
97	AIRTRSGGNTNQQKA	4
98	RSGGNTNQQKASAGQ	4
99	NTNQQKASAGQISVQ	4
100	QKASAGQISVQPTFS	4
101	AGQISVQPTFSVQRN	4
102	SVQPTFSVQRNLPFE	4
103	TFSVQRNLPFERATV	4
104	QRNLPFERATVMAAF	4
105	PFERATVMAAFSGNN	4
106	ATVMAAFSGNNEGRT	4
107	AAFSGNNEGRTSDMR	4
108	GNNEGRTSDMRTEVI	4
109	GRTSDMRTEVIRMME	4
110	DMRTEVIRMMESAKP	4
111	EVIRMMESAKPEDLS	4
112	MMESAKPEDLSFQGR	4
113	AKPEDLSFQGRGVFE	4
114	DLSFQGRGVFELSDE	4
115	QGRGVFELSDEKATN	4
116	VFELSDEKATNPIVP	4
117	SDEKATNPIVPSFDM	4
118	ATNPIVPSFDMSNEG	4
119	IVPSFDMSNEGSYFF	4
120	FDMSNEGSYFFGDNA	4
121	NEGSYFFGDNAEEYD	4
122	YFFGDNAEEYDN	4

Linear pools		
Pool	# peptides	which peptides
Pool 1	25	1-25
Pool 2	30	26-55
Pool 3	34	56-89
Pool 4	33	90-122

B

pVACC-NP^{H3} Peptides

Peptide ID	Peptide Sequence	Pool #
1	SQGTKRSYEQMETDG	1
2	KRSYEQMETDGDRQN	1
3	EQMETDGDRQNATEI	1
4	TDGDRQNATEIRASV	1
5	RQNATEIRASVGKMI	1
6	TEIRASVGKMIDGIG	1
7	ASVGKMIDGIGRFYI	1
8	KMIDGIGRFYIQMCT	1
9	GIGRFYIQMCTELKL	1
10	FYIQMCTELKLSDHE	1
11	MCTELKLSDHEGRLI	1
12	LKLSDHEGRLIQNSL	1
13	DHEGRLIQNSLTIEK	1
14	RLIQNSLTIEKMVLS	1
15	NSLTIEKMVLSAFDE	1
16	IEKMVLSAFDERRNK	1
17	VLSAFDERRNKYLEE	1
18	FDERRNKYLEEHPSA	1
19	RNKYLEEHPSAGKDP	1
20	LEEHPSAGKDPKKTG	1
21	PSAGKDPKKTGGPIY	1
22	KDPKKTGGPIYRRVD	1
23	KTGGPIYRRVDGKWM	1
24	PIYRRVDGKWMRELV	1
25	RVDGKWMRELVLYDK	1

Peptide ID	Peptide Sequence	Pool #
26	KWMRELVLYDKEEIR	2
27	ELVLYDKEEIRRIWR	2
28	YDKEEIRRIWRQANN	2
29	EIRRIWRQANNGEDA	2
30	IWRQANNGEDATSGL	2
31	ANNGEDATSGLTHIM	2
32	EDATSGLTHIMIWHS	2
33	SGLTHIMIWHSNLND	2
34	HIMIWHSNLNDATYQ	2
35	WHSNLNDATYQRTRA	2
36	LNDATYQRTRALVRT	2
37	TYQRTRALVRTGMDP	2
38	TRALVRTGMDPRMCS	2
39	VRTGMDPRMCSLMQG	2
40	MDPRMCSLMQGSTLP	2
41	MCSLMQGSTLPRRSG	2
42	MQGSTLPRRSGAAGA	2
43	TLPRRSGAAGAAVKG	2
44	RSGAAGAAVKGIGTM	2
45	AGAAVKGIGTMVMEL	2
46	VKGIGTMVMELIRMV	2
47	GTMVMELIRMVKRGI	2
48	MELIRMVKRGINDRN	2
49	RMVKRGINDRNFWRG	2
50	RGINDRNFWRGENGR	2
51	DRNFWRGENGRKTRS	2
52	WRGENGRKTRSAYER	2
53	NGRKTRSAYERMCNI	2
54	TRSAYERMCNILKGK	2
55	YERMCNILKGKFQTA	2

Peptide ID	Peptide Sequence	Pool #
56	CNILKGKFQTAAQRA	3
57	KGKFQTAAQRAMVDQ	3
58	QTAAQRAMVDQVRES	3
59	QRAMVDQVRESRNPG	3
60	VDQVRESRNPGNAEI	3
61	RESRNPGNAEIEDLI	3
62	NPGNAEIEDLIFLAR	3
63	AEIEDLIFLARSALI	3
64	DLIFLARSALILRGS	3
65	LARSALILRGSVAHK	3
66	ALILRGSVAHKSCLP	3
67	RGSVAHKSCLPACAY	3
68	AHKSCLPACAYGPAV	3
69	CLPACAYGPAVSSGY	3
70	CAYGPAVSSGYDFEK	3
71	PAVSSGYDFEKEGYS	3
72	SGYDFEKEGYSLVGI	3
73	FEKEGYSLVGIDPFK	3
74	GYSLVGIDPFKLLQN	3
75	VGIDPFKLLQNSQIY	3
76	PFKLLQNSQIYSLIR	3
77	LQNSQIYSLIRPNEN	3
78	QIYSLIRPNENPAHK	3
79	LIRPNENPAHKSQLV	3
80	NENPAHKSQLVWMAC	3
81	AHKSQLVWMACHSAA	3
82	QLVWMACHSAAFEDL	3
83	MACHSAAFEDLRLLS	3
84	SAAFEDLRLLSFIRG	3
85	EDLRLLSFIRGTKVS	3
86	LLSFIRGTKVSPRGK	3
87	IRGTKVSPRGKLSTR	3
88	KVSPRGKLSTRGVQI	3
89	RGKLSTRGVQIASNE	3

Peptide ID	Peptide Sequence	Pool #
90	STRGVQIASNENMDN	4
91	VQIASNENMDNMGSS	4
92	SNENMDNMGSSTLEL	4
93	MDNMGSSTLELRSGY	4
94	GSSTLELRSGYWAIR	4
95	LELRSGYWAIRTRSG	4
96	SGYWAIRTRSGGNTN	4
97	AIRTRSGGNTNQQRA	4
98	RSGGNTNQQRASAGQ	4
99	NTNQQRASAGQTSVQ	4
100	QRASAGQTSVQPTFS	4
101	AGQTSVQPTFSVQRN	4
102	SVQPTFSVQRNLPFE	4
103	TFSVQRNLPFEKSTI	4
104	QRNLPFEKSTIMAAF	4
105	PFEKSTIMAAFTGNT	4
106	STIMAAFTGNTEGRT	4
107	AAFTGNTEGRTSDMR	4
108	GNTEGRTSDMRAEII	4
109	GRTSDMRAEIIRMME	4
110	DMRAEIIRMMEGAKP	4
111	EIIRMMEGAKPEEVS	4
112	MMEGAKPEEVSFRGR	4
113	AKPEEVSFRGRGVFE	4
114	EVSFRGRGVFELSDE	4
115	RGRGVFELSDEKATN	4
116	VFELSDEKATNPIVP	4
117	SDEKATNPIVPSFDM	4
118	ATNPIVPSFDMSNEG	4
119	IVPSFDMSNEGSYFF	4
120	FDMSNEGSYFFGDNA	4
121	NEGSYFFGDNAEEYD	4
122	YFFGDNAEEYDN	4

Linear pools		
Pool	# peptides	which peptides
Pool 1	25	1-25
Pool 2	30	26-55
Pool 3	34	56-89
Pool 4	33	90-122

C

Peptide Matrix Pool Design

Pools	1	2	3	4	5	6	7	8	9	10	11	12
13	1	2	3	4	5	6	7	8	9	10	11	12
14	13	14	15	16	17	18	19	20	21	22	23	24
15	25	26	27	28	29	30	31	32	33	34	35	36
16	37	38	39	40	41	42	43	44	45	46	47	48
17	49	50	51	52	53	54	55	56	57	58	59	60
18	61	62	63	64	65	66	67	68	69	70	71	72
19	73	74	75	76	77	78	79	80	81	82	83	84
20	85	86	87	88	89	90	91	92	93	94	95	96
21	97	98	99	100	101	102	103	104	105	106	107	108
22	109	110	111	112	113	114	115	116	117	118	119	120
23	121	122										
